# Improved overall survival in patients with high-grade serous ovarian cancer is associated with CD16a+ immunologic neighborhoods containing NK cells, T cells and macrophages

**DOI:** 10.3389/fimmu.2023.1307873

**Published:** 2024-01-22

**Authors:** Sarah Nersesian, Riley J. Arseneau, Jorge P. Mejia, Stacey N. Lee, Lauren P. Westhaver, Nigel W. Griffiths, Stephanie R. Grantham, Liliane Meunier, Laudine Communal, Avik Mukherjee, Anne-Marie Mes-Masson, Thomas Arnason, Brad H. Nelson, Jeanette E. Boudreau

**Affiliations:** ^1^ Department of Microbiology and Immunology, Dalhousie University, Halifax, NS, Canada; ^2^ Beatrice Hunter Cancer Research Institute, Halifax, NS, Canada; ^3^ Department of Pathology, Dalhousie University, Halifax, NS, Canada; ^4^ Centre de recherche du Centre hospitalier de l’Université de Montréal and Institut du cancer de Montréal, Montreal, QC, Canada; ^5^ Akoya Biosciences, Menlo Park, CA, United States; ^6^ Department of Medicine, Université de Montréal, Montreal, QC, Canada; ^7^ Department of Pathology & Laboratory Medicine, QEII Health Sciences Centre, Nova Scotia Health (Central Zone), Halifax, NS, Canada; ^8^ Deeley Research Centre, British Columbia Cancer Research Institute, Victoria, BC, Canada; ^9^ Department of Biochemistry and Microbiology, University of Victoria, Victoria, BC, Canada; ^10^ Department of Medical Genetics, University of British Columbia, Vancouver, BC, Canada

**Keywords:** natural killer (NK) cell, ovarian cancer, high grade serous cancer, spatial biology, CD16A

## Abstract

**Background:**

For patients with high grade serous carcinoma of the ovary (HGSC), survival rates have remained static for the last half century. Despite the presence of tumor mutations and infiltration of immune cells, existing immunotherapies have achieved little success against HGSC. These observations highlight a gap in the understanding of how the immune system functions and interacts within HGSC tumors.

**Methods:**

We analyzed duplicate core samples from 939 patients with HGSC to understand patterns of immune cell infiltration, localization, and associations with clinical features. We used high-parameter immunohistochemical/Opal multiplex, digital pathology, computational biology, and multivariate analysis to identify immune cell subsets and their associations with HGSC tumors.

**Results:**

We defined six patterns of cellular infiltration by spatially restricted unsupervised clustering of cell subsets. Each pattern was represented to some extent in most patient samples, but their specific distributions differed. Overall (OS) and progression-free survival (PFS) corresponded with higher infiltration of CD16a^+^ cells, and their co-localization with macrophages, T cells, NK cells, in one of six cellular neighborhoods that we defined with our spatial assessment.

**Conclusions:**

Immune cell neighborhoods containing CD16a+ cells are associated with improved OS and PFS for patients with HGSC. Patterns of immunologic neighborhoods differentiate patient outcomes, and could inform future, more precise approaches to treatment.

## Introduction

High grade serous carcinoma of the ovary (HGSC) is the most common, aggressive, genetically unstable, and fatal form of ovarian cancer (1–3). Lack of effective screening methods and specific early symptoms typically lead to late-stage diagnosis: 80% of patients are diagnosed at stage III or IV, when the disease has already metastasized (1, 2). HGSC tumors initially respond to a combination of platinum- and taxane-based chemotherapy, but over 70% recur, and second-line treatments aim primarily at prolonging life rather than curing disease (2, 4). Overall survival (OS) in patients with HGSC corresponds with infiltration of immune cells in the tumor, but a better understanding of the interactions that facilitate anti-cancer immunity are required to maximize immune-mediated cancer control.

Despite the presence of intra-tumoral immune cells and an intermediate neoantigen load (5, 6), anti-PD-L1 therapies have been largely ineffective against HGSC, with an overall response rate of 10-15% (7–12). T cells (13), macrophages (14, 15), plasma cells (16), and NK cells (17) infiltrating HGSC, especially the epithelial regions (18), have each been associated with improved prognosis for patients. Although individual cells can be prognostic in isolation, the generation and execution of immune responses requires collaboration between different immune cell types. For instance, activation of T cell responses against a tumor requires HLA presentation of a tumor antigen, alongside the requisite co-stimulation and cytokines (19). Since defects in components of the HLA processing and presentation pathway and antigen escape variants are common in cancers, lymphocytes like NK cells that act independently of these are key players in comprehensive tumor control (20, 21). Finally, antibody production by plasma cells requires help from activated effector T cells, and additional cellular mediators to carry out antibody-dependent functions.

Antibody-dependent cellular cytotoxicity (ADCC) is facilitated by binding of the CD16a receptor (FCGR3A) to the fragment-crystallizable (Fc) region of an IgG antibody (22), which activates cytotoxic killing of antibody-bound targets. Expression of the CD16a isoform is typically restricted to natural killer (NK) cells and monocytes/macrophages, though there are reports of its expression by other immune populations in specific disease contexts, including conventional and non-conventional T cells (23, 24) and fibroblasts (25). The presence of CD16a^+^ immune cells in the tumor associates with greater sensitivity to chemotherapy and longer progression-free survival (PFS) and overall survival in patients with recurrent HGSC (25), suggesting a role for CD16a^+^ cells in tumor control.

Immunity is multifaceted, and interactions between immune cell subtypes might be important considerations in defining prognosis and designing future immunotherapies. With spatial biology approaches, patterns of immune cell infiltration – “cellular neighborhoods” – provide proof of this concept. For example, enrichment of PD-1^+^CD4^+^ T cells within granulocyte rich neighborhoods is associated with survival in a high-risk of patients with colorectal cancer (26). In patients with HGSC, spatial transcriptomics and single cell RNA sequencing followed by computational clustering differentiate responders and non-responders to neoadjuvant chemotherapy and long versus short term survivors (27, 28). These studies have identified prognostic features that represent anti-tumor immunity, and therefore, additional investigation to understand immune mechanisms and to modify their function toward anti-tumor immunity is warranted.

We designed multiplex immunofluorescence panels to simultaneously identify HGSC epithelium, T cells, macrophages, NK cells, and CD16a^+^ cells in an HGSC tissue microarray (TMA). Using digital pathology and spatial analyses, we quantified immune cell density, distribution into epithelial and stromal compartments, and spatial proximity to other cells. We find that CD68^+^CD16a^+^ cells are associated with longer OS, and co-localize in the HGSC microenvironment in non-random patterns. With unsupervised clustering, we define major “cellular neighborhoods”, including two enriched for CD16a^+^ cells. A higher ratio of area covered by CD16a+ enriched neighborhoods, especially when they contained NK cells, T cells and macrophages, to neighborhoods lacking immune infiltration also associated with longer OS and PFS. Our data collectively imply that CD16a+ cells mark neighborhoods of immune cells that may co-operate to mount effective immunity against HGSC.

## Methods

### Patients and tissue microarrays

TMA slides were obtained after scientific review from the COEUR (The Canadian Ovarian Experimental Unified Resource) committee. Patient samples included in the TMA were collected from 12 Canadian ovarian cancer biobanks, from 1992 to 2014, and detailed patient demographics and tumor characteristics have been published (29) (A summary of these cohorts is available in **Supplementary Table 1**). The COEUR TMA (TMA A) consists of duplicate core tumors isolated from 1,159 patients with HGSC. Following tissue staining, image processing, and pathologist review, 939 patients were analyzed. The second TMA (TMA B) represented a subset of patients, n=24. All methods for specimens and clinical information collection and subsequent analyses were approved by Dalhousie University’s Research Ethics Board (#2020-5060). The use of primary peripheral blood mononuclear cells (PBMC) to validate antibodies was approved by the Dalhousie University REB (#2016-3842) and the Canadian Blood Services REB (#2016-016) and collected in collaboration with the Canadian Blood Services Blood4Research program.

### Opal multiplex immunofluorescence

**Figure 1:**
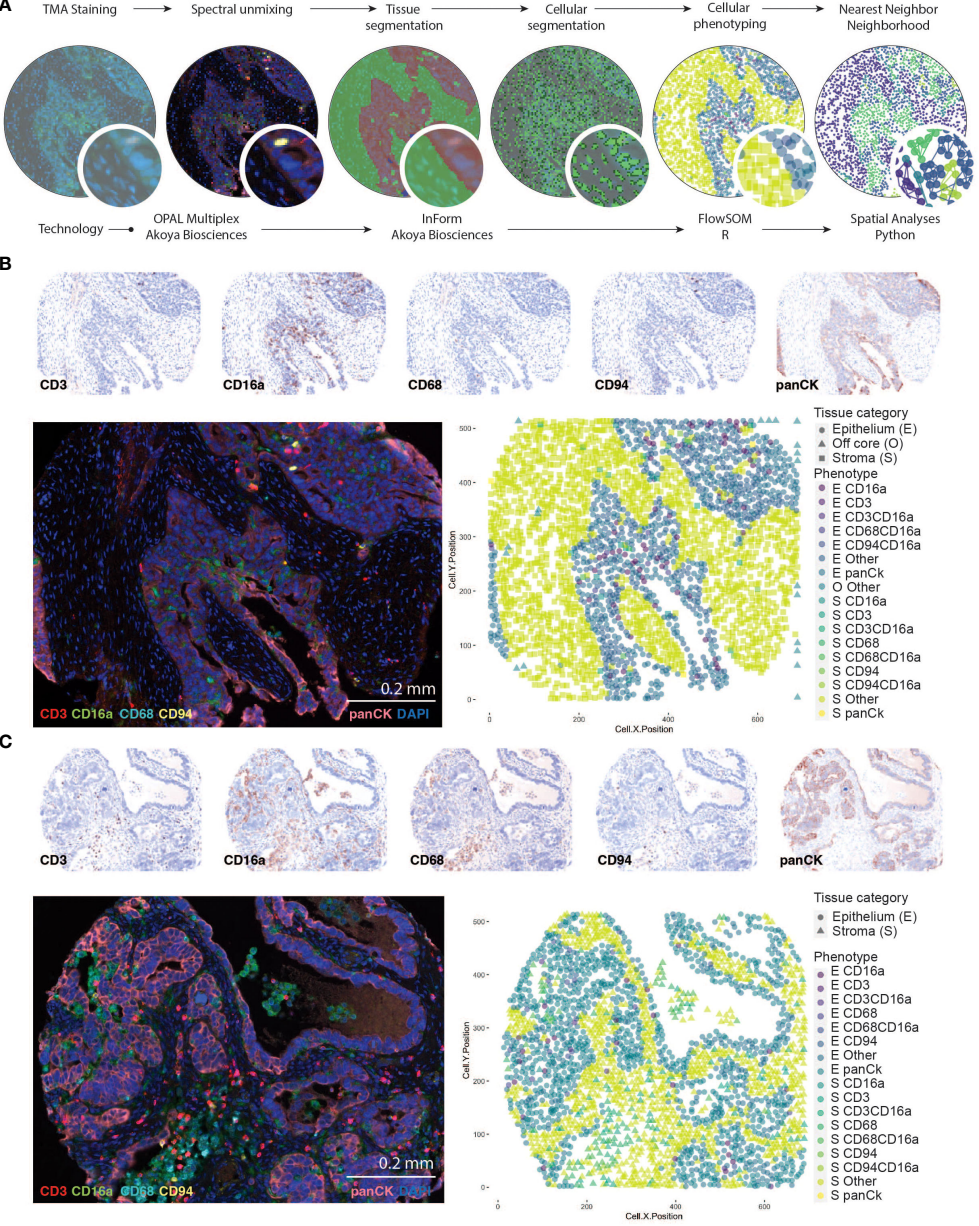
Identifying CD16a+ cell populations in HGSC. **(A)** Schematic representation of analysis workflow steps (top labels) and analysis (bottom labels). TMA staining is conducted with Opal multiplexing (staining and imaging), followed by spectral unmixing to enable visualization of each marker independently. Tissue segmentation (based on anti-Pan-cytokeratin staining) segregates the epithelial and stromal tumor compartments, and cell segmentation (facilitated by DAPI nuclear staining) enables identification of individual cells. Cell phenotypes are trained with a board-certified pathologist (TA), and automatically counted using InForm software, which includes geographic coordinates. Cells are thereafter counted and analyzed with FlowSOM in RStudio; nearest neighbor neighborhood analysis is conducted using the Spatial algorithm in Python. **(B, C)** Representative images of an HGSC tumor with immune cell infiltration in the **(B)** epithelial or **(C)** stromal compartments of the tumor. Individual signals from multispectral images were used to produce “pathology-view” pseudo-stained images. Images represent individual markers that together represent the multiplex staining as imaged in the fluorescent micrograph. The fluorescent images represent pseudo-colored micrographs taken at 20X magnification (scale bar = 0.2mm). Computationally reconstructed phenotype map after tissue and cellular segmentation and cellular phenotyping.

Our multiplex immunofluorescence protocol was established in accordance with the Society for Immunotherapy of Cancer’s best practices for multiplex immunohistochemistry and immunofluorescence staining and validation (30) and we used equipment, software, and reagents from Akoya Biosciences unless otherwise indicated. Antibody staining was validated using cell pellets created from primary PBMC (**Supplementary Figure 1**). CD56 is the antibody expected to best identify NK cells in tissues, but staining for CD56 in HGSC is complicated because it is overexpressed in a subset of tumors. To circumvent this challenge, we instead used anti-CD94, which identifies cells expressing the NKG2A or NKG2C receptor found on most NK cells (**Supplementary Figure 2**). TMA slides were de-paraffinized, rehydrated, and fixed in 10% neutral buffered formalin for 25 min. Antigen retrieval was conducted by microwave treatment (2 min at 100% power followed by 15 min at 20% power, 1000W microwave) in Tris-EDTA buffer (pH 9). Slides were cooled for 15 min at room temperature, then rinsed with deionized water, Tris-Buffered Saline, and Tween-20 (TBS-T) buffer. The tyramide signal amplification (TSA)-based IF staining protocol was conducted according to the Opal 7-color manual IHC kit. Slides were incubated in blocking buffer for 10 min to stabilize epitopes and reduce background staining. Slides were then incubated with a primary antibody for 45 min, rinsed in TBS-T, and incubated with secondary anti-mouse and anti-rabbit HRP for 10 min. Slides were rinsed and Fluorophore staining was then conducted with an Opal fluorophore in Opal amplification diluent for 10 min. The slides were then rinsed and underwent a second microwave treatment to remove the previous antibody. Steps were sequentially repeated for each antibody in the multiplex panel [Panel 1: (CD3 (LN10, 1/50), CD16a (SP175, 1/50), CD94 (ERP21003, 1/100), CD68 (SP251, 1/100), panCK (AE1/AE3, 1/50)] [Panel 2: CD163 (ERP19518, 1/100), CD68 (SP251, 1/100), CD94 (ERP21003, 1/50), panCK (AE1/AE3, 1/50), CD16a (SP175, 1/50), CD8 (C8/144, pre-diluted)] (**Supplementary Table 2**). The order of antibody staining was optimized empirically, and generally occurred in the order from most to least frequent epitope in the panel. Following staining of the last antibody, slides were incubated with Spectral DAPI for 5 min, rinsed and mounted in Prolong Gold mounting media (Invitrogen). Cell surface specific antibody binding patterns were validated by a board-certified pathologist (TA) (**Figure 1**; **Supplementary Figure 3**).

### Multispectral analysis and automated quantitative pathology

Images were captured using a PhenoImager Quantitative Pathology workstation with multispectral separation capabilities, at 10 nm wavelength intervals from 420 nm to 740 nm, and 20x magnification. Images were visualized and processed in InForm Tissue Finder Software to conduct multispectral analysis (extracting fluorescent signatures) and subsequent quantitative pathology (**Figure 1**). Monoplexed stains were used to calibrate spectral imaging by creating a spectral reference library. Following spectral unmixing, tissues were virtually segmented based on pan-cytokeratin staining and autofluorescence to define regions as tumor epithelia, tumor stroma, vasculature/autofluorescence. This was followed by cellular segmentation to identified individual cell nuclei based on DAPI staining. Each cell was then assigned a unique cell ID which was used to quantify the antibody signal surrounding the individual nuclei and establish X,Y coordinates. These antibody signals were used to phenotype cells using FlowSOM in R Studio (version 4.2) where cells were phenotyped into unique clusters. Specifically, the fluorescent signals (markers) from each cell X,Y coordinate were extracted and underwent log normalization, followed by transformation into a flow frame using the ‘DFtoFF’ function available within the FlowSOM package. Elbow plots were generated to established the optimal number of clusters. Unsupervised clustering was then conducted using the ‘FlowSOM’ function with no scaling and optimal cluster number: panel 1 = 43, panel 2 = 40). Following this, FlowSOM defined clusters were re-assigned as one of the following phenotypes based on their expression of markers; panCK^+^ were identified as epithelial cells (regardless of expression of other markers), panCK^-^CD68^+^ cells were identified as the myeloid population (+/-CD16a), panCK^-^CD68^-^CD3^+^ cells were identified as T cells (+/-CD94+/- CD16a), panCK^-^CD68^-^CD3^-^CD94^+^ cells were identified as NK cells (+/- CD16a), panCK^-^CD68^-^CD3^-^CD94^-^CD16a^+^ were identified as CD16a+ cells and cells negative for all markers were identified as Other.

### Spatial analyses

Spatial analysis was conducted using Python version 3.10.9 with the following packages: matplotlib (v.3.6.3), NumPy (v.1.22.4), pandas (v.1.5.3), scikit-learn (v.1.2.2), and seaborn (v.0.12.2). This analysis was based on the bioinformatics approach described previously (26) using input data that was generated and exported from our automated quantitative pathology in InForm. This file contained individual cell IDs, their corresponding patient sample ID, X/Y coordinates, and reported phenotypes.

For each individual cell in each sample, we quantified the phenotypes of the closest “k” (5, 10, 15 or 20) neighboring cells based on their X/Y coordinates using the scikit-learn NearestNeighbors module. The result was a data frame that included the initial data and additional columns with counts for each neighboring phenotype. Next, we employed MiniBatchKMeans to cluster cells into “n” (5-10) neighborhoods based on the phenotype counts of their neighboring cells. To evaluate the composition of each neighborhood and select the optimal “k” and “n” values to avoid under or over fitting, we graphed in a heatmap the enrichment of individual phenotypes in each neighborhood. We performed multiple combinations of this analysis by using different values of “n” and “k”. To select the final values, we examined similarities between neighborhoods in the same run and in previous iterations. Optimal “k” and “n” were considered when there were no highly similar neighborhoods in the same run and if reducing the values merged two clusters of interest. After defining the final number of neighborhoods, we generated tables containing the most frequent neighboring cells for each phenotype, the frequency of neighborhoods for each patient, and plots showing the spatial distribution of neighborhoods in each sample.

### Statistical analysis

GraphPad Prism 9 and R Studio were used to generate graphically visualized data, and R Studio was used to conduct Cox regression survival analyses. Both univariate and multivariate Cox regression analyses were conducted to determine significant differences in survival between various groups. High and low infiltrated tumors were defined based on median infiltration density (cells/mm^2^) of each immune cell subset, and quartiles were defined for categorical Cox regressions. Differences in infiltration numbers between groups categorized by clinical parameters were determined by one or two-way ANOVAs correcting for false discovery rates and presenting as adjusted p-values using the “fdr” method from the p.adjust() function in R Studio (Version 4.2.2), as appropriate. Chemosensitivity was defined by responsiveness at six months post-treatment. To evaluate the co-infiltration between intratumoral immune cells or neighborhoods, a Pearson’s correlation was conducted with all r values listed for each pair in the correlation matrix. The threshold for statistical significance was set at alpha = 0.05; however, non-statistical but clinically relevant associations, defined by the authors, were described along with corresponding *p* values.

## Results

### HGSC tissue microarray and patient characteristics

We conducted multiplex immunofluorescence staining and quantitative analysis on a formalin-fixed paraffin-embedded (FFPE) HGSC tissue microarray (TMA) containing duplicate samples from 1,159 patients. After staining, quality control, and removal of patients who histologically classified as having non-HGSC tumors, 939 patients could be analyzed (886 treatment naïve, 53 chemotherapy-treated; **Supplementary Table 1**). We first conducted univariate analysis to define whether tumor stage, tumor grade, debulking status, age, or *BRCA1/2* mutation status were independently associated with survival outcomes among treatment-naïve patients (**Table 1**; **Supplementary Table 3**). Lower tumor stage, surgical debulking status, and younger age at diagnosis were significant predictors of OS, and lower tumor stage and surgical debulking status were also significant predictors of PFS. Neither age, grade (2 vs 3) nor *BRCA1/2* mutation status were significant predictors of PFS in this cohort (**Table 1**).

**Table 1:** Cox proportional hazards analysis for PFS and OS.

Univariate analysis	Progression-free survival				Overall survival			
Clinical Variables	HR	95% CI	*p*	*Adj. p*	HR	95% CI	*p*	*Adj. p*
Age (older vs. younger patients)	1.00	[0.997, 1.01]	0.234	0.234	1.02	[1.01, 1.03]	<0.0001	<0.0001
Stage (high stage vs. low stage)	1.93	[1.68, 2.22]	<0.0001	<0.0001	1.76	[1.48, 2.11]	<0.0001	<0.0001
Debulking status (not debulked vs. successfully debulked)	2.18	[1.80, 2.64]	<0.0001	<0.0001	2.48	[1.95, 3.15]	<0.0001	<0.0001
Immune Infiltration (categorical variable, +/- median)								
Stroma CD68CD16A	0.869	[0.739, 1.02]	0.0913	0.0913	0.799	[0.653, 0.978]	0.0295	0.0295
Epithelium CD68CD16A	0.869	[0.738, 1.02]	0.0902	0.0902	0.782	[0.639, 0.958]	0.0177	0.0177
Neighborhood proportion (categorical variable, +/- mean)								
CD16A enriched epithelium	0.835	[0.710, 0.982]	0.0297	0.0297	0.781	[0.639, 0.955]	0.0160	0.0160
CD16A enriched stroma	0.850	[0.723, 1.00]	0.0499	0.0499	0.850	[0.695, 1.04]	0.111	0.111
Immune Infiltration (categorical variable, Q1/Q4)								
Stroma CD16A	0.777	[0.618, 0.977]	0.0307	0.0307	0.688	[0.515, 0.920]	0.0115	0.0115
Stroma CD68CD16A	0.828	[0.657, 1.05]	0.113	0.113	0.715	[0.539, 0.948]	0.0199	0.0199
Stroma PanCK	1.10	[0.813, 1.28]	0.399	0.399	1.34	[1.01, 1.78]	0.0407	0.0407
Epithelium CD16A	0.802	[0.636, 1.01]	0.0645	0.0645	0.753	[0.563, 1.01]	0.0556	0.0556
Neighborhood proportion (categorical variable, +/- mean) (n=24)								
N6	0.588	[0.238, 1.45]	0.250	0.250	0.242	[0.0748, 0.785]	0.0181	0.0181

Statistically significant Cox proportional hazards analysis are shown for progression-free survival (PFS) and overall survival (OS) for 886 treatment-naïve patients based on clinical characteristics, infiltrating immune cells and area covered by immune cell neighborhoods.

To identify immune cell populations in HGSC with spatial resolution, we developed a novel Opal multiplex IF panel and analysis workflow (**Figure 1**). To circumvent challenges associated with epithelial expression of CD56 in HGSC that have been reported previously (31), we included and validated staining for CD94, a protein that heterodimerizes with natural killer group-2 (NKG2) family members, which are present on most NK cells (**Supplementary Figure 2**). To separate the two canonical subsets of NK cells, we included staining for CD16a, a receptor associated with the CD56^dim^ cytolytic population and performance of antibody-dependent cellular cytotoxicity (ADCC) (32). We also included CD3 and CD68 to identify T cells and macrophages, respectively. Automated tissue segmentation (i.e., epithelium vs stroma) was highly concordant with the pathologist (TA) assessment (**Supplementary Figure 3**). Automated cellular segmentation slightly undercounted (~84% of actual) cells in epithelial regions and overcounted (~108% of actual) in stromal regions (**Supplementary Figure 3**).

**Figure 2:**
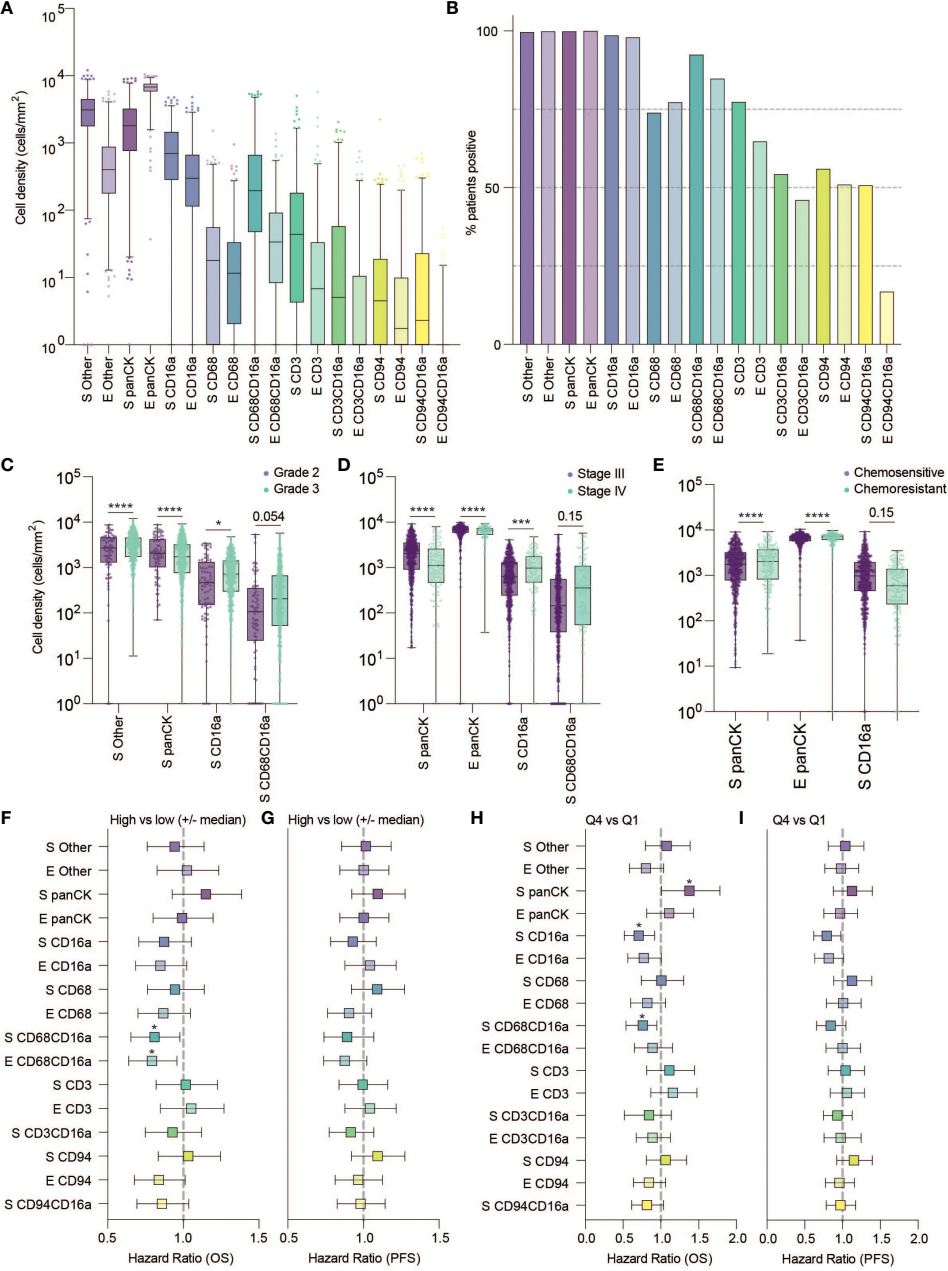
Frequency, clinical associations, and hazard ratios based on independent cell populations and localization in HGSC. **(A)** Cell density in the stromal and epithelial compartments of HGSC tumor cores (E, epithelium; S, stromal). **(B)** Percentage of patients positive for each cell population. **(C–E)** Cell densities of statistically significant cell population based on tumor characteristics: **(C)** grade **(D)** Stage and **(E)** Chemo-sensitivity at six months (statistical significance determined by two-way ANOVA controlling for false discovery rates, adjusted p-values are represented by ****p < 0.0001, ***p < 0.001, *p < 0.05). **(F–H)** Forrest plots representing the hazard ratio based on univariate Cox proportional hazard models. Plots show 95% confidence interval comparing **(F, G)** high (above the median) versus low (below the median) infiltration, or **(H, I)** the highest quartile compared to the lowest quartile. Survival outcomes represent **(F, H)** five-year overall survival (OS) or **(G, I)** five-year progression free survival (*adjusted p < 0.05).

After spectral unmixing, digital pathology and unsupervised clustering, we quantified each cell population separately in the intrastromal (S) or intraepithelial (E) regions of tumor cores (**Figures 2A, B**). Of the immune cell populations evaluated, CD16a^+^ cells were the most abundant subset: almost every tumor on the TMA contained at least some CD16a+ cells (CD16a^+^CD68^-^CD3^-^CD94^-^). The second-most abundant cell population was macrophages (CD68^+^CD16a^-^CD3^-^CD94^-/+^ and CD68^+^CD16a^+^CD3^-^CD94^-/+^), followed by T cells (CD3^+^CD16a^-^CD68^-^CD94^-/+^ and CD3^+^CD16a^+^CD68^-^CD94^-/+^), and NK cells (CD94^+^CD16a^-^CD3^-^CD68^-^ and CD94^+^ CD16a^+^CD3^-^CD68^-^). All immune cells were present at higher frequencies within the stromal compartment compared with the epithelial compartment.

We next considered the relationship between infiltrating cell densities and clinical characteristics including tumor grade, stage, and chemotherapy response (chemo-sensitive or -resistant at six months). Compared with grade 2 tumors, grade 3 tumors were more infiltrated with CD16a^+^ cells, while exhibiting no significant differences in CD16a^-^ immune populations (**Figure 2C**). Tumor stage was also significantly correlated with immune cell densities: we observed significantly higher densities of CD16a^+^, and CD68^+^CD16a^+^ cells in the stromal compartments of stage IV compared with stage III HGSC tumors (**Figure 2D**). No significant associations were observed for infiltrating immune cell density comparing chemo-sensitive to chemo-resistant patients, however stromal CD16a^+^ cells demonstrated a trend towards an decrease in chemo-resistant patients (p=0.15) (**Figure 2E**). We also observed significant associations with OS when comparing the high vs low (around the median) immune cell density of epithelial or stromal CD68^+^CD16a^+^ and the top and bottom quartiles of stromal CD68^+^CD16a^+^ or CD16a^+^ cells (**Figures 2F–I**; **Table 1**). When comparing the tumor cells themselves, we observed significantly shorter OS when panCK^+^ cells were present in the stromal regions. These associations remained after controlling for other significant variables in multivariate Cox regression (**Table 2**). Altogether, these findings demonstrate that CD16a^+^ cells are abundant within HGSC, their density increases as a tumor progresses, and their localization and presence is associated with survival outcomes.

**Table 2:** Multivariate Cox proportional hazards analysis for PFS and OS in patients for variables defined as significant in univariate analysis.

Multivariate analysis	Progression-free survival				Overall survival			
Variables	HR	95% CI	*p*	*Adj. p*	HR	95% CI	*p*	*Adj. p*
Stroma CD68CD16A					0.671	[0.490, 0.918]	0.0127	0.0212
Epithelium CD68CD16A					0.652	[0.476, 0.894]	0.0078	0.0149
CD16a enriched epithelium	0.822	[0.637, 1.06]	0.131	0.218	0.660	[0.483, 0.906]	0.00999	0.0166
CD16a enriched stroma	0.816	[0.630, 1.06]	0.124	0.207				
(+/- Q1/Q4)								
Stroma PanCK					1.63	[1.11, 2.85]	0.0168	0.0392
Stroma CD16A					0.488	[0.305, 0.781]	0.00279	0.00976
Stroma CD68CD16A					0.666	[0.428, 1.04]	0.0719	0.126

### CD16a^+^ immune cells form distinct cellular neighborhoods within HGSC tumors

**Figure 3:**
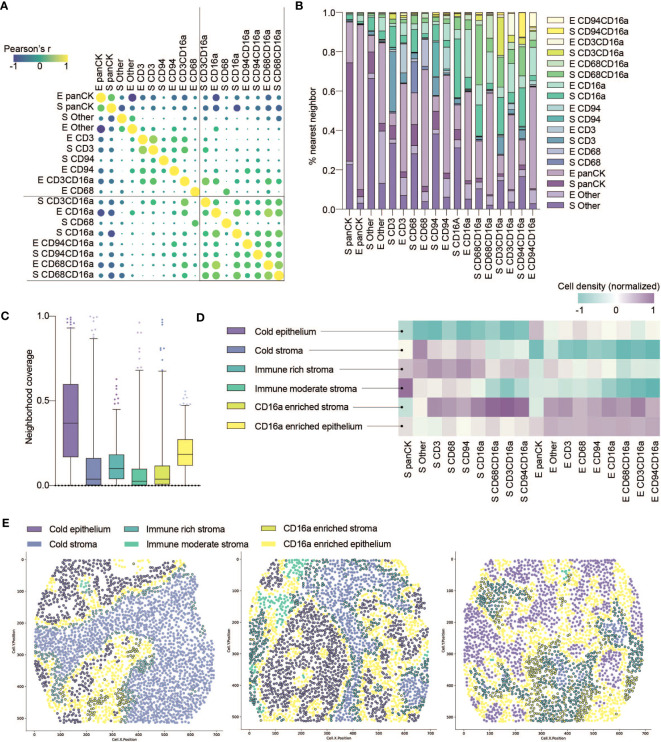
Co-infiltration, colocalization and spatial cell clustering into cellular neighborhoods within HGSC tumor cores. **(A)** Co-infiltration of cell densities within HGSC cores as determined by Pearson’s correlation matrix. Strength of association is represented by color scale and size of dot. **(B)** Percent co-localization of each cell phenotype with another as determined by nearest neighbor analysis (k=15). **(C)** Median neighborhood coverage among all HGSC tumor cores. **(D)** Heatmap depicting the density of each individual cell phenotype in each of the spatially defined cellular neighborhoods. **(E)** Three representative computationally reconstructed HGSC tumor cores and their spatial distribution of cellular neighborhoods.

A co-ordinated immune response likely requires both co-infiltration (co-incident presence) and co-localization (close proximity) of immune cells in the same tumor. We used Pearson’s correlations to assess co-infiltration, examining the densities of cell subsets for each patient, averaging between their duplicate cores. Regardless of tumor region, CD16a^+^ cells co-infiltrated with each other, but rarely with CD16a^-^ immune cell populations (**Figure 3A**).

That immune cell infiltration appeared to be non-random prompted us to consider how they co-localize into spatially-defined cellular neighborhoods. We first quantified the nearest neighbors for cells from each population, and observed that CD16a^+^ cells indeed spatially co-localize within HGSC cores (**Figure 3B**). Notably, CD16a^-^ cells do not show a similarly high degree of proximity to CD16a^+^ cells, indicating that the effect is not merely due to the relative abundance of CD16a^+^ cells. We next conducted spatial analyses to cluster cell subsets into multicellular neighborhoods, using unsupervised clustering to consider the 15 nearest neighbors of each cell. This revealed six unique cellular neighborhoods that varied in their frequency, distribution, and cellular composition between patients (**Figures 3C, D**). Two of these neighborhoods were devoid of immune infiltration and predominantly either epithelial or stromal; we refer to them as (i) cold epithelium and (ii) cold stroma, respectively. The remaining four neighborhoods were immune infiltrated; among them, two were highly infiltrated with CD16a^+^ cells: (iii) CD16a enriched stroma and (iv) CD16a enriched epithelium, and the remaining two neighborhoods were defined based on the extent of immune cell infiltration, as (v) immune-rich or (vi) immune-moderate (**Figure 3D**).

We observed patterns of cellular neighborhoods’ localization in cores (**Figure 3E**). CD16a enriched neighborhoods (stromal and epithelial) had similar collections of cell subtypes. In some patients, cells were mostly localized to the stromal regions (CD16a enriched stroma), while others had these cells in both the stromal and epithelial regions (CD16a enriched epithelium). CD16a enriched neighborhoods were mostly found at the junction between the stromal and epithelial regions. The majority of cold epithelium was found within “tumor nests” (regions of epithelial cells alone). Similarly, cold stromal regions were often distanced from the immune cell neighborhoods, representing regions of tumors where immune cells are excluded.

### CD16a enriched cellular neighborhoods are associated with better OS and PFS

**Figure 4:**
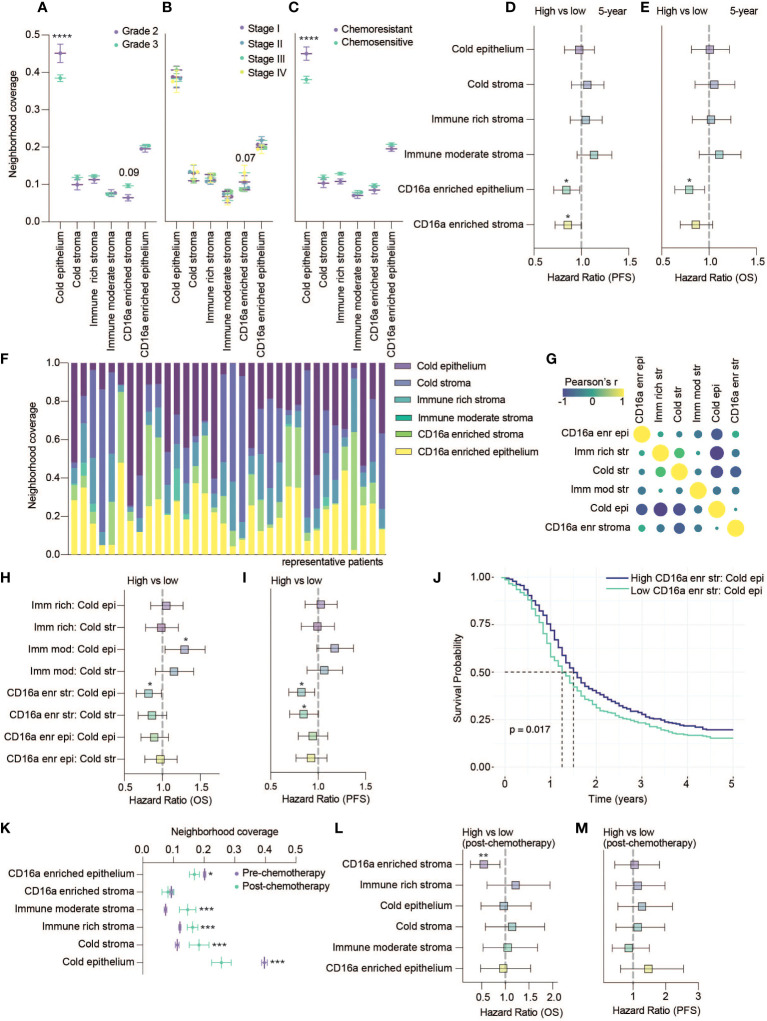
CD16a enriched cellular neighborhoods are associated with better OS and PFS. **(A–C)** Neighborhood coverage proportion of HGSC tumor cores based on tumor characteristics: **(A)** Grades, **(B)** Stages and **(C)** Chemo-sensitivity (statistical significance determined by two-way ANOVA, controlling for false discovery rates, adjusted p-values are represented by ****p < 0.0001). **(D, E)** Forrest plots representing univariate Cos proportional hazard ratios, 95% confidence interval for the high compared to low neighborhood coverage, split based on median, for **(D)** five-year PFS survival outcomes or **(E)** five-year OS (*p < 0.05). **(F)** Proportion of cellular neighborhood coverage across a random set of representative patients. **(G)** Co-occupancy of cellular neighborhoods determined by Pearson’s correlation matrix. **(H, I)** Forrest plots representing univariate Cox proportional hazard ratios, 95% confidence interval for the high compared to low ratios of immune rich, immune moderate or CD16a enriched neighborhoods for **(H)** five-year OS or **(I)** five-year PFS (*p < 0.05). **(J)** Kaplan-Meier survival plot comparing the PFS of high vs low ratio of CD16a enriched stroma to cold epithelium. **(K)** Neighborhood coverage in a HGSC tumor cores isolated from patients pre- vs post- chemotherapy at the time of sample collection (statistical significance determined by one-way ANOVA between all neighborhoods, *p < 0.05, ***p < 0.001). Forrest plots representing univariate Cox proportional hazard ratios, 95% confidence interval for the high compared to low neighborhood coverage, for **(L)** five-year OS or **(M)** five-year PFS (**p < 0.01).

To consider the relevance of the six immune cell neighborhoods we defined, we explored their associations with clinical variables. Compared with grade 2 tumors, grade 3 tumors were generally infiltrated with more immune cells (**Figure 2C**), grade 3 tumors were occupied by more CD16a enriched stroma (p = 0.09) and significantly less cold epithelium (**Figure 4A**). We found CD16a enriched stroma was the only neighborhood to differ between stage III and IV tumors (p = 0.07), with stage IV tumors having higher proportions of CD16a^+^ cells (**Figure 4B**). Neighborhoods were also associated with chemotherapy response: tumors resistant to chemotherapy exhibited greater ratios of cold epithelium (**Figure 4C**). Chemo-sensitive patients also exhibited a non-significant trend toward larger areas of CD16a enriched neighborhoods compared with chemo-resistant patients. A larger area of both CD16a enriched stroma and epithelium predicted better PFS (**Figure 4D**) and a larger area of CD16a enriched epithelial neighborhood predicted better OS (**Figure 4E**; **Table 1**). This association with OS remained for CD16a enriched epithelium when controlling for other significant variables by multivariate Cox regression analysis (**Table 2**). Taken together, these findings suggest that co-operation between leukocyte subsets occurs in CD16a+ cell regions, where NK cells, T cells, and macrophages co-localize.

Each cell neighborhood was present in nearly all patients, but their relative abundances differed (**Figure 4F**). Cold epithelium was inversely correlated with CD16a enriched stroma, immune rich stroma, and cold stroma (**Figure 4G**). We found that greater ratios of CD16a enriched stroma:cold epithelium were associated with improved PFS and OS, and greater CD16a enriched stroma:cold stroma was associated with longer PFS. However, a greater ratio of immune moderate stroma:cold epithelium was associated with worse OS (**Figures 4H–J**). These findings highlight that proximal associations of specific cell types (neighborhoods) – especially around CD16a+ cells – is predictive of outcomes for patients with HGSC, not simply immune cell infiltration alone.

A small subset of patients (n=53) were treated with chemotherapy prior to surgery. We excluded these patients from earlier analyses but used these patients to investigate whether chemotherapy treatment influences the proportion of cellular neighborhoods. We found that the area of epithelial neighborhoods (infiltrated and non-infiltrated) was lower in post-chemotherapy patients, and stromal neighborhoods were enriched (**Figure 4K**). We observed a decrease in the area of CD16a enriched epithelial neighborhoods in patients that received chemotherapy (**Figure 4K**). When evaluating if any neighborhoods associated with survival, we again found that CD16a enriched stromal cellular neighborhoods are associated with better OS (**Figures 4L, M**). In sum, and despite a smaller cohort, our findings suggest that chemotherapy could impact the distribution of cell neighborhoods in patients with HGSC.

### CD8^+^ cells colocalize in CD16a enriched neighborhoods and together predict clinical outcomes

**Figure 5:**
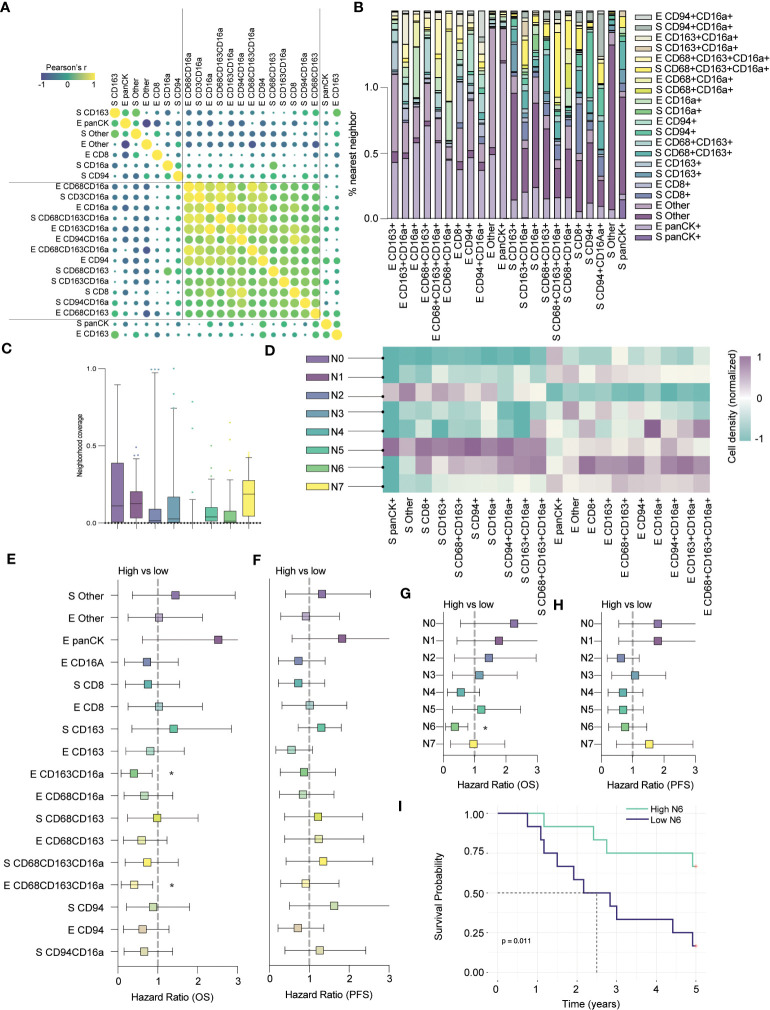
CD8+ cells colocalize in CD16a enriched neighborhoods and together predict clinical outcomes. **(A)** Co-infiltration of cell densities within HGSC cores as determined by Pearson’s correlation. The strength of each association is represented by color scale and size of dot. **(B)** Percent co-localization of cell subtypes as determined by nearest neighbor analysis. **(C)** Median neighborhood coverage among HGSC tumor cores. **(D)** Heatmap depicting the density of each individual cell phenotype in each of the spatially defined cellular neighborhoods. **(E–H)** Forrest plots representing univariate Cox proportional hazard ratios, 95% confidence interval for the high compared to low for **(E)** five-year overall survival (OS) or **(F)** five-year progression free survival (*p < 0.05). Forrest plots representing the hazard ratio, result from univariate Cox proportional hazard models, +/- 95% confidence interval for the high compared to low neighborhood coverage, split based on median, against survival outcomes **(G)** five-year OS or **(H)** PFS (*p < 0.05). **(I)** Kaplan-Meier survival plot comparing the PFS of high vs low N6 neighborhood coverage within HGSC tumor cores.

Previous studies have demonstrated a prognostic benefit of T cells, but our assessment of T cells alone, using anti-CD3, did not yield significant impact on OS or PFS. Reasoning that more specifically identifying T cell and macrophage subsets might better highlight prognostic differences, we expanded our panel to include anti-CD8 and anti-CD163. CD8^+^ T cells are generally cytotoxic and associated with beneficial outcomes; conversely, CD163^+^ macrophages are associated with immune regulation and poorer outcomes (14, 33). We stained cores from a subset of 24 patients of the original cohort and found that while most infiltrating immune cell types co-infiltrated HGSC tumors, stromal CD163+ and epithelial CD8+ cells were not as strongly correlated with other immune populations (**Figure 5A**). When looking at nearest neighbors within this panel, we again observed that CD16a^+^ cells co-localize with each other (**Figure 5B**).

Next, we applied our spatial algorithm to define distinct cellular neighborhoods and generated eight unique clusters, which we termed N0 – N7 (**Figure 5C**). The output of this analysis resembled our earlier clustering; N0 and N3 mirrored the cold epithelium, and N2 mirrored the cold stroma (**Figure 5D**). We also observed immune (non-CD16a) enriched neighborhoods, in the stroma (N1) and epithelium (N7), as in our previous analyses (similar to immune-moderate and immune-rich, respectively). Finally, we found clusters that mirrored the CD16a enriched neighborhoods: N4 was highly enriched for epithelial CD16a^+^ and CD68^+^CD163^+^CD16a+ cells, while N5 was enriched for broad immune populations but localized to the stroma. N6 most closely mirrored our CD16a enriched epithelial neighborhood and notably, this neighborhood was also enriched for stromal and epithelial CD8^+^ cells.

Next, we evaluated how cell infiltration or proportion of neighborhoods within the cores correlated to an exploratory survival analysis. Although we examined only 24 cases in this subset analysis, we found that epithelial infiltration by CD163^+^CD16a^+^ cells or CD68^+^CD163^+^CD16a^+^ cells were favorably prognostic for overall survival, while cells expressing CD163^+^ or CD68^+^CD163^+^ were not (**Figures 5E–G**). In terms of neighborhood proportion, N4 approached a survival benefit but was not statistically significant (**Figures 5E–G**). Only N6 (CD16a and CD8 enriched epithelium) predicted OS, (p=0.011, **Figures 5G, I**), but not PFS (**Figure 5H**). In sum, the presence of immune-enriched neighborhoods, especially those with CD16a^+^ cells, is prognostically beneficial.

## Discussion

New and effective treatments for HGSC are urgently needed, but the ideal configuration and activities of immune cells to combat these tumors is not yet clear. We studied immunologic features of 939 patients with HGSC using a TMA; most were treatment-naïve. We used digital pathology to interrogate the distribution and spatial relationships of intratumoral immune cell populations, quantifying cells known to associate with outcomes in patients with HGSC: macrophages, T cells, and NK cells and testing their infiltration and localization for associations with clinical characteristics. Nearly all patients had some immune cell infiltration in their tumors, and the intrastromal presence of CD16a^+^ cells – which can potentially facilitate ADCC and ADCP (34) – is associated with improved survival. We find that immune cells spatially cluster into non-random cellular “neighborhoods”, which are found to occupy varying area in HGSC tumors. Patients with leukocytes organized into “CD16a^+^ enriched” neighborhoods (which contain CD16a^+^ cells, NK cells, T cells, and macrophages), had the longest OS and PFS of any group, suggesting that a co-ordinated immune response might facilitate the best tumor growth control. Our results provide insight into how spatial analysis of tumor samples may inform prognosis, and highlights a beneficial immune configuration for immunotherapies.

In recurrent HGSC, and primary colorectal carcinoma, CD16a^+^ infiltrating immune cells have been previously associated with OS and PFS by univariate analysis (25, 35), but whether these cells are relevant, and why, has not been studied in treatment-naïve patients with HGSC. We confirm and extend this finding here: CD16a+ cell infiltration predicts longer OS for patients with HGSC, even after controlling for other significant variables, including tumor stage where CD16a cell density is elevated in stage IV compared to stage III HGSC. A limitation of our work is that we cannot conclude the specific cellular identity of the single positive CD16a^+^ population, and that it likely represents more than one type of cell (**Supplementary Figure 4**). CD16a is the key antibody receptor leading to ADCC, involved in ADCP, and, when activated, its downstream signaling can promote cytokine release for further immune cell activation (34). CD16a is primarily expressed on subsets of NK cells, macrophages, and monocytes, and can be present and functional on other cellular populations including cancer-associated fibroblasts (36) and subsets of T cells (23, 24, 37). Indeed, we observed CD16a frequently being co-expressed on cells positive for CD68 and CD3 (**Supplementary Figure 5**). CD16a expression in macrophages, including tumor-associated macrophages, has been associated with an “M1”-like phenotype, which promotes inflammation via cytokine release and antigen presentation (38). Finally, any cell that has the capacity for trogocytosis could take up CD16a and maintain its expression on the cell surface with or without downstream functionality depending on the cell type (39, 40).

Immune mechanisms rarely act in isolation, and for CD16a, ADCC and ADCP require bound antibodies, which are produced by plasma cells activated with help from T cells (41). Indeed, future studies exploring ADCC and ADCP should consider their interaction with plasma cells or available anti-tumor antibodies. In this study, and others, infiltration of each of CD8^+^ T cells, macrophages, plasma cells, and CD57^+^ NK cells into HGSC were associated with improved survival for patients (17). Here, we find CD16a^+^ cells, macrophages, NK cells, and T cells often co-infiltrate and co-localize in the same cores. This likely reflects shared trafficking or signals for immune cell infiltration into tumors – a feature that might be exploited to design immunotherapies.

Since immune cells co-operate, cellular co-localization could be an indicator of immune activity in the tumor. With an unsupervised analysis, we identified six patterns of immune cell colocalization that were defined by the proximity and ratios of cell subsets. Interestingly, most patients had representation from each the neighborhoods in their tumors, but the relative area represented by these neighborhoods varied. With additional markers, we found that greater area represented by N6 neighborhoods which contain the highest proportions of CD8^+^, CD94^+^, and CD16a^+^CD94 in the tumor epithelium, was associated with longer OS and PFS. Noteworthy, larger representation of this population by area is associated with responsiveness to chemotherapy, suggesting that this configuration is more amenable to effective treatment.

We observed both CD8^+^ and CD163^+^ cells in CD16a+ cellular neighborhoods. While cytotoxic CD8^+^ T cells are well-established to correspond with beneficial outcomes, we were surprised to record a benefit of CD163^+^CD16a^+^ cells, since the CD163 marker is typically associated with M2 macrophages and poorer anti-cancer functions (14). Nevertheless, CD163^+^CD16a^+^ cells have been previously described in a group of HGSC patients, and to associate with superior outcomes with high densities of both CD3^+^ and CD163^+^ immune populations (42). Perhaps CD163^+^ cells represent a dysfunctional or exhausted macrophage population that lost CD16 expression, as has been reported in breast cancer (43) and other pathologies (44). Additional studies are warranted to fully characterize this population of cells and explore its responsiveness to therapy or ways to reinvigorate it for anti-cancer function.

We were able to assess the impact of platinum chemotherapy in a subset of 53 patients. Prior treatment with any chemotherapy resulted in a shift from high epithelial neighborhood proportions towards more stromal neighborhood proportions and a decrease in the CD16a^+^ enriched epithelium, confirming the value of CD16a enriched stromal neighborhoods persists even after chemotherapy. Although we observed statistical significance with this small subset of patients, we did not have repeat sampling on the same patients, so further investigation will be needed to define whether these alterations are directly prompted by chemotherapy.

Most of our patients were treatment-naïve at the time of sampling, and usually went on to receive standard chemotherapy. The associations that we observe – where immune neighborhoods are associated with improved survival – reflect patient’s outcomes after treatment. Our results therefore imply that there is an “ideal” immunologic configuration for patients with HGSC treated with existing and conventional therapy, but also endorse strategies to understand how existing and nascent immunotherapies shape the immune contexture of tumors, develop immunotherapies that drive formation of productive immune cell neighborhoods, or stratify treatment based on the immunologic contexture of a patient’s tumor. These may include opportunities to prevent CD16a cleavage with matrix metalloproteinase inhibitors, or cellular therapies with non-cleavable CD16a^+^ molecules (45–47). Immunologic modifiers, such as immune checkpoint inhibitors that rescue leukocytes from inhibition and anergy, or bi- and tri-specific engagers that force interactions between key immune populations could change the immune contexture to make patients’ tumors more amenable for immune recognition (48–50).

Here, we identified that CD16a-expressing immune cells and their spatial localization within HGSC tumors is associated with distribution and localization of other immune cells, and together, these neighborhoods of cells can be prognostic markers for patient outcomes in HGSC and may help stratify responders from non-responders to therapeutic agents. Colocalization of CD16a^+^ and CD8^+^ cells suggests a potential synergistic effect that promotes anti-tumor immunity. It is likely that there are additional features of each of these, and other immune cell populations that are informative, and will help to refine the definition of an “ideal” immune configuration.

## Data availability statement

The raw data supporting the conclusions of this article will be made available by the authors, without undue reservation.

## Ethics statement

All methods for specimens and clinical information collection and subsequent analyses were approved by Dalhousie University’s Research Ethics Board (#2020-5060). The use of primary peripheral blood mononuclear cells (PBMC) to validate antibodies was approved by the Dalhousie University REB (#2016-3842) and the Canadian Blood Services REB (#2016-016) and collected in collaboration with the Canadian Blood Services Blood4Research program. The studies were conducted in accordance with the local legislation and institutional requirements. All human samples were from the Terry Fox Research Institue COEUR cohort. Informed patient consent was obtained prior to sample collection. The study of this cohort is approved by the Comité d’éthique de la recherche from the Centre hospitalier de l’Université de Montréal (#09.141). Written informed consent for participation was not required from the participants or the participants’ legal guardians/next of kin in accordance with the national legislation and institutional requirements.

## Author contributions

SN: Conceptualization, Data curation, Formal analysis, Investigation, Methodology, Validation, Visualization, Writing – original draft, Writing – review & editing. RA: Data curation, Formal analysis, Methodology, Writing – original draft, Writing – review & editing. JM: Data curation, Formal analysis, Methodology, Writing – original draft, Writing – review & editing. SL: Data curation, Investigation, Methodology, Writing – review & editing. LW: Data curation, Investigation, Writing – original draft, Writing – review & editing. NG: Resources, Writing – review & editing. SG: Data curation, Formal analysis, Methodology, Writing – original draft, Writing – review & editing. LM: Methodology, Resources, Writing – review & editing. LC: Methodology, Resources, Writing – review & editing. AM: Methodology, Resources, Writing – review & editing. AM-M: Methodology, Resources, Writing – review & editing. TA: Formal analysis, Methodology, Validation, Writing – review & editing. BN: Methodology, Resources, Writing – review & editing. JB: Conceptualization, Funding acquisition, Methodology, Project administration, Resources, Supervision, Writing – original draft, Writing – review & editing.

## References

[1] Bast RC Jr. Hennessy B Mills GB. The biology of ovarian cancer: new opportunities for translation. Nat Rev Cancer (2009) 9(6):415–28. doi: 10.1038/nrc2644 PMC2814299 19461667

[2] Torre LA Trabert B DeSantis CE Miller KD Samimi G Runowicz CD. Ovarian cancer statistics, 2018. CA Cancer J Clin (2018) 68(4):284–96. doi: 10.3322/caac.21456 PMC6621554 29809280

[3] Pugh-Toole M Nicolela AP Nersesian S Leung BM Boudreau JE. Natural killer cells: the missing link in effective treatment for high-grade serous ovarian carcinoma. Curr Treat Options Oncol (2022) 23:210–26. doi: 10.1007/s11864-021-00929-x 35192139

[4] du Bois A Reuss A Pujade-Lauraine E Harter P Ray-Coquard I Pfisterer J. Role of Surgical Outcome as Prognostic Factor in Advanced Epithelial Ovarian Cancer: A Combined Exploratory Analysis of 3 Prospectively Randomized Phase 3 Multicenter Trials By the Arbeitsgemeinschaft Gynaekologische Onkologie Studiengruppe Ovarialkarzinom (AGO-OVAR) and the Groupe d'Investigateurs Nationaux Pour les Etudes des Cancers de l'Ovaire (GINECO). Cancer-Am Cancer Soc (2009) 115(6):1234–44. doi: 10.1002/cncr.24149 19189349

[5] Jimenez-Sanchez A Cybulska P Mager KL Koplev S Cast O Couturier DL. Unraveling tumor-immune heterogeneity in advanced ovarian cancer uncovers immunogenic effect of chemotherapy. Nat Genet (2020) 52(6):582–93. doi: 10.1038/s41588-020-0630-5 PMC8353209 32483290

[6] Matsushita H Hasegawa K Oda K Yamamoto S Asada K Karasaki T. Neoantigen load and HLA-class I expression identify a subgroup of tumors with a T-cell-inflamed phenotype and favorable prognosis in homologous recombination-proficient high-grade serous ovarian carcinoma. J Immunother Cancer (2020) 8(1):1–11. doi: 10.1136/jitc-2019-000375 PMC7254153 32461346

[7] Clouthier DL Lien SC Yang SYC Nguyen LT Manem VSK Gray D. An interim report on the investigator-initiated phase 2 study of pembrolizumab immunological response evaluation (INSPIRE). J Immunother Cancer (2019) 7(1):72. doi: 10.1186/s40425-019-0541-0 30867072 PMC6417194

[8] Matulonis UA Shapira-Frommer R Santin AD Lisyanskaya AS Pignata S Vergote I. Antitumor activity and safety of pembrolizumab in patients with advanced recurrent ovarian cancer: results from the phase II KEYNOTE-100 study. Ann Oncol (2019) 30(7):1080–7. doi: 10.1093/annonc/mdz135 31046082

[9] Varga A Piha-Paul S Ott PA Mehnert JM Berton-Rigaud D Morosky A. Pembrolizumab in patients with programmed death ligand 1-positive advanced ovarian cancer: Analysis of KEYNOTE-028. Gynecol Oncol (2019) 152(2):243–50. doi: 10.1016/j.ygyno.2018.11.017 30522700

[10] Hamanishi J Mandai M Ikeda T Minami M Kawaguchi A Murayama T. Safety and antitumor activity of anti-PD-1 antibody, nivolumab, in patients with platinum-resistant ovarian cancer. J Clin Oncol (2015) 33(34):4015–22. doi: 10.1200/JCO.2015.62.3397 26351349

[11] Liu JF Gordon M Veneris J Braiteh F Balmanoukian A Eder JP. Safety, clinical activity and biomarker assessments of atezolizumab from a Phase I study in advanced/recurrent ovarian and uterine cancers. Gynecol Oncol (2019) 154(2):314–22. doi: 10.1016/j.ygyno.2019.05.021 31204078

[12] Disis ML Taylor MH Kelly K Beck JT Gordon M Moore KM. Efficacy and safety of avelumab for patients with recurrent or refractory ovarian cancer: phase 1b results from the JAVELIN solid tumor trial. JAMA Oncol (2019) 5(3):393–401. doi: 10.1001/jamaoncol.2018.6258 30676622 PMC6439837

[13] Wang W Zou W Liu JR. Tumor-infiltrating T cells in epithelial ovarian cancer: predictors of prognosis and biological basis of immunotherapy. Gynecol Oncol (2018) 151(1):1–3. doi: 10.1016/j.ygyno.2018.09.005 30243508 PMC6263945

[14] Hensler M Kasikova L Fiser K Rakova J Skapa P Laco J. M2-like macrophages dictate clinically relevant immunosuppression in metastatic ovarian cancer. J Immunother Cancer (2020) 8(2):1–12. doi: 10.1136/jitc-2020-000979 PMC7443306 32819974

[15] Corvigno S Mezheyeuski A de la Fuente LM Westbom-Fremer S Carlson JW Fernebro J. High density of stroma-localized CD11c-positive macrophages is associated with longer overall survival in high-grade serous ovarian cancer. Gynecol Oncol (2020) 159(3):860–8. doi: 10.1016/j.ygyno.2020.09.041 33032823

[16] Garsed DW Pandey A Fereday S Kennedy CJ Takahashi K Alsop K. The genomic and immune landscape of long-term survivors of high-grade serous ovarian cancer. Nat Genet (2022) 54(12):1853–64. doi: 10.1038/s41588-022-01230-9 PMC10478425 36456881

[17] Henriksen JR Donskov F Waldstrom M Jakobsen A Hjortkjaer M Petersen CB. Favorable prognostic impact of Natural Killer cells and T cells in high-grade serous ovarian carcinoma. Acta Oncol (2020) 59(6):652–9. doi: 10.1080/0284186X.2019.1711173 31931651

[18] Webb JR Milne K Nelson BH. Location, location, location: CD103 demarcates intraepithelial, prognostically favorable CD8(+) tumor-infiltrating lymphocytes in ovarian cancer. Oncoimmunology (2014) 3:e27668. doi: 10.4161/onci.27668 25101220 PMC4121334

[19] van der Leun AM Thommen DS Schumacher TN. CD8(+) T cell states in human cancer: insights from single-cell analysis. Nat Rev Cancer (2020) 20(4):218–32. doi: 10.1038/s41568-019-0235-4 PMC7115982 32024970

[20] de Vries NL van de Haar J Veninga V Chalabi M Ijsselsteijn ME van der Ploeg M. gammadelta T cells are effectors of immunotherapy in cancers with HLA class I defects. Nature (2023) 613(7945):743–50. doi: 10.1038/s41586-022-05593-1 PMC9876799 36631610

[21] Malmberg KJ Sohlberg E Goodridge JP Ljunggren HG. Immune selection during tumor checkpoint inhibition therapy paves way for NK-cell "missing self" recognition. Immunogenetics (2017) 69(8-9):547–56. doi: 10.1007/s00251-017-1011-9 PMC5537320 28699110

[22] Petricevic B Laengle J Singer J Sachet M Fazekas J Steger G. Trastuzumab mediates antibody-dependent cell-mediated cytotoxicity and phagocytosis to the same extent in both adjuvant and metastatic HER2/neu breast cancer patients. J Transl Med (2013) 11:307. doi: 10.1186/1479-5876-11-307 24330813 PMC4029549

[23] Bjorkstrom NK Gonzalez VD Malmberg KJ Falconer K Alaeus A Nowak G. Elevated numbers of Fc gamma RIIIA+ (CD16+) effector CD8 T cells with NK cell-like function in chronic hepatitis C virus infection. J Immunol (2008) 181(6):4219–28. doi: 10.4049/jimmunol.181.6.4219 18768879

[24] Clemenceau B Vivien R Debeaupuis E Esbelin J Biron C Levy Y. FcgammaRIIIa (CD16) induction on human T lymphocytes and CD16pos T-lymphocyte amplification. J Immunother (2011) 34(7):542–9. doi: 10.1097/CJI.0b013e31822801d4 21760529

[25] Lalos A Neri O Ercan C Wilhelm A Staubli S Posabella A. High density of CD16+ Tumor-infiltrating immune cells in recurrent ovarian cancer is associated with enhanced responsiveness to chemotherapy and prolonged overall survival. Cancers (Basel) (2021) 13(22):1:13. doi: 10.3390/cancers13225783 PMC8616362 34830938

[26] Schurch CM Bhate SS Barlow GL Phillips DJ Noti L Zlobec I. Coordinated cellular neighborhoods orchestrate antitumoral immunity at the colorectal cancer invasive front. Cell (2020) 183(3):838. doi: 10.1016/j.cell.2020.07.005 33125896 PMC7658307

[27] Stur E Corvigno S Xu M Chen K Tan Y Lee S. Spatially resolved transcriptomics of high-grade serous ovarian carcinoma. iScience (2022) 25(3):103923. doi: 10.1016/j.isci.2022.103923 35252817 PMC8891954

[28] Ferri-Borgogno S Zhu Y Sheng J Burks JK Gomez JA Wong KK. Spatial transcriptomics depict ligand-receptor cross-talk heterogeneity at the tumor-stroma interface in long-term ovarian cancer survivors. Cancer Res (2023) 83(9):1503–16. doi: 10.1158/0008-5472.CAN-22-1821 PMC10159916 36787106

[29] Le Page C Rahimi K Kobel M Tonin PN Meunier L Portelance L. Characteristics and outcome of the COEUR Canadian validation cohort for ovarian cancer biomarkers. BMC Cancer (2018) 18(1):347. doi: 10.1186/s12885-018-4242-8 29587661 PMC5872529

[30] Taube JM Akturk G Angelo M Engle EL Gnjatic S Greenbaum S. The Society for Immunotherapy of Cancer statement on best practices for multiplex immunohistochemistry (IHC) and immunofluorescence (IF) staining and validation. J Immunother Cancer (2020) 8(1):1–14. doi: 10.1136/jitc-2019-000155corr1 PMC7239569 32414858

[31] Bösmüller HC Wagner P Pham DL Fischer AK Greif K Beschorner C. CD56 (Neural cell adhesion molecule) expression in ovarian carcinomas: association with high-grade and advanced stage but not with neuroendocrine differentiation. Int J Gynecol Cancer (2017) 27(2):239–45. doi: 10.1097/IGC.0000000000000888 27984374

[32] Caligiuri MA. Human natural killer cells. Blood (2008) 112(3):461–9. doi: 10.1182/blood-2007-09-077438 PMC2481557 18650461

[33] Ovarian Tumor Tissue Analysis C Goode EL Block MS Kalli KR Vierkant RA Chen W. Dose-response association of CD8+ Tumor-infiltrating lymphocytes and survival time in high-grade serous ovarian cancer. JAMA Oncol (2017) 3(12):e173290. doi: 10.1001/jamaoncol.2017.3290 29049607 PMC5744673

[34] Coenon L Villalba M. From CD16a biology to antibody-dependent cell-mediated cytotoxicity improvement. Front Immunol (2022) 13:913215. doi: 10.3389/fimmu.2022.913215 35720368 PMC9203678

[35] Sconocchia G Zlobec I Lugli A Calabrese D Iezzi G Karamitopoulou E. Tumor infiltration by FcgammaRIII (CD16)+ myeloid cells is associated with improved survival in patients with colorectal carcinoma. Int J Cancer (2011) 128(11):2663–72. doi: 10.1002/ijc.25609 PMC3426287 20715106

[36] Liu X Lu Y Huang J Xing Y Dai H Zhu L. CD16(+) fibroblasts foster a trastuzumab-refractory microenvironment that is reversed by VAV2 inhibition. Cancer Cell (2022) 40(11):1341–57.e13. doi: 10.1016/j.ccell.2022.10.015 36379207

[37] Yeap WH Wong KL Shimasaki N Teo EC Quek JK Yong HX. CD16 is indispensable for antibody-dependent cellular cytotoxicity by human monocytes. Sci Rep (2016) 6:34310. doi: 10.1038/srep34310 27670158 PMC5037471

[38] Shi Y Fan X Deng H Brezski RJ Rycyzyn M Jordan RE. Trastuzumab triggers phagocytic killing of high HER2 cancer cells in *vitro* and in *vivo* by interaction with Fcgamma receptors on macrophages. J Immunol (2015) 194(9):4379–86. doi: 10.4049/jimmunol.1402891 25795760

[39] Daubeuf S Lindorfer MA Taylor RP Joly E Hudrisier D. The direction of plasma membrane exchange between lymphocytes and accessory cells by trogocytosis is influenced by the nature of the accessory cell. J Immunol (2010) 184(4):1897–908. doi: 10.4049/jimmunol.0901570 20089699

[40] Dance A. Core Concept: Cells nibble one another via the under-appreciated process of trogocytosis. Proc Natl Acad Sci USA (2019) 116(36):17608–10. doi: 10.1073/pnas.1912252116 PMC6731757 31481628

[41] Cao X Chen J Li B Dang J Zhang W Zhong X. Promoting antibody-dependent cellular phagocytosis for effective macrophage-based cancer immunotherapy. Sci Adv (2022) 8(11):eabl9171. doi: 10.1126/sciadv.abl9171 35302839 PMC8932662

[42] Ardighieri L Missale F Bugatti M Gatta LB Pezzali I Monti M. Infiltration by CXCL10 secreting macrophages is associated with antitumor immunity and response to therapy in ovarian cancer subtypes. Front Immunol (2021) 12:690201. doi: 10.3389/fimmu.2021.690201 34220848 PMC8253056

[43] Medrek C Ponten F Jirstrom K Leandersson K. The presence of tumor associated macrophages in tumor stroma as a prognostic marker for breast cancer patients. BMC Cancer (2012) 12:306. doi: 10.1186/1471-2407-12-306 22824040 PMC3414782

[44] Magalhaes GM Guimaraes CF Paula MC. Case for diagnosis. Patch granuloma annulare Bras Dermatol (2017) 92(3):419–20. doi: 10.1590/abd1806-4841.20176729 PMC5514593 29186265

[45] Zhu H Blum RH Bjordahl R Gaidarova S Rogers P Lee TT. Pluripotent stem cell-derived NK cells with high-affinity noncleavable CD16a mediate improved antitumor activity. Blood (2020) 135(6):399–410. doi: 10.1182/blood.2019000621 31856277 PMC7005364

[46] Cichocki F Bjordahl R Goodridge JP Mahmood S Gaidarova S Abujarour R. Quadruple gene-engineered natural killer cells enable multi-antigen targeting for durable antitumor activity against multiple myeloma. Nat Commun (2022) 13(1):7341. doi: 10.1038/s41467-022-35127-2 36446823 PMC9709157

[47] Cichocki F Goodridge JP Bjordahl R Mahmood S Davis ZB Gaidarova S. Dual antigen-targeted off-the-shelf NK cells show durable response and prevent antigen escape in lymphoma and leukemia. Blood (2022) 140(23):2451–62. doi: 10.1182/blood.2021015184 PMC9918847 35917442

[48] Gleason MK Ross JA Warlick ED Lund TC Verneris MR Wiernik A. CD16xCD33 bispecific killer cell engager (BiKE) activates NK cells against primary MDS and MDSC CD33+ targets. Blood (2014) 123(19):3016–26. doi: 10.1182/blood-2013-10-533398 PMC4014844 24652987

[49] Vallera DA Felices M McElmurry R McCullar V Zhou X Schmohl JU. IL15 trispecific killer engagers (TriKE) make natural killer cells specific to CD33+ Targets while also inducing persistence, *in vivo* expansion, and enhanced function. Clin Cancer Res (2016) 22(14):3440–50. doi: 10.1158/1078-0432.CCR-15-2710 PMC4947440 26847056

[50] Floerchinger A Klein JE Finkbeiner MSC Schafer TE Fuchs G Doerner J. A vector-encoded bispecific killer engager to harness virus-activated NK cells as anti-tumor effectors. Cell Death Dis (2023) 14(2):104. doi: 10.1038/s41419-023-05624-3 36765035 PMC9918448

