# Supplementary material for: Improved overall survival in patients with high-grade serous ovarian cancer is associated with CD16a+ immunologic neighborhoods containing NK cells, T cells and macrophages
Source: Front Immunol. 2024 Jan 22;14:1307873. doi: 10.3389/fimmu.2023.1307873 (PMC10838965; doi:10.3389/fimmu.2023.1307873)
Supplement: Supplementary file 1 [file DataSheet_1.docx]

**SUPPLEMENTAL MATERIAL**

**Supplementary Figure 1: Validation of antibody staining. A)** NK and T cells were purified from healthy donor PBMCs and prepared as pellets, fixed and mounted in FFPE, then used to test antibody binding specificity. Green indicates target cell staining and cells are counterstained with DAPI (blue). **B)** Antibody dilutions on tonsil tissue. Green boxes indicate the concentration used in multiplex analysis. Pink staining indicates antibody target and cells are counterstained with DAPI. **C)** Staining of each markers alone (left) or in combination (right) with the multiplex panel on test samples of HGSC tissue, for the indicates antibodies. All cells are counterstained with DAPI. Images were captured on a PhenoImager Quantitative Pathology workstation at 20x magnification. PanCk, pan-cytokeratin.

*
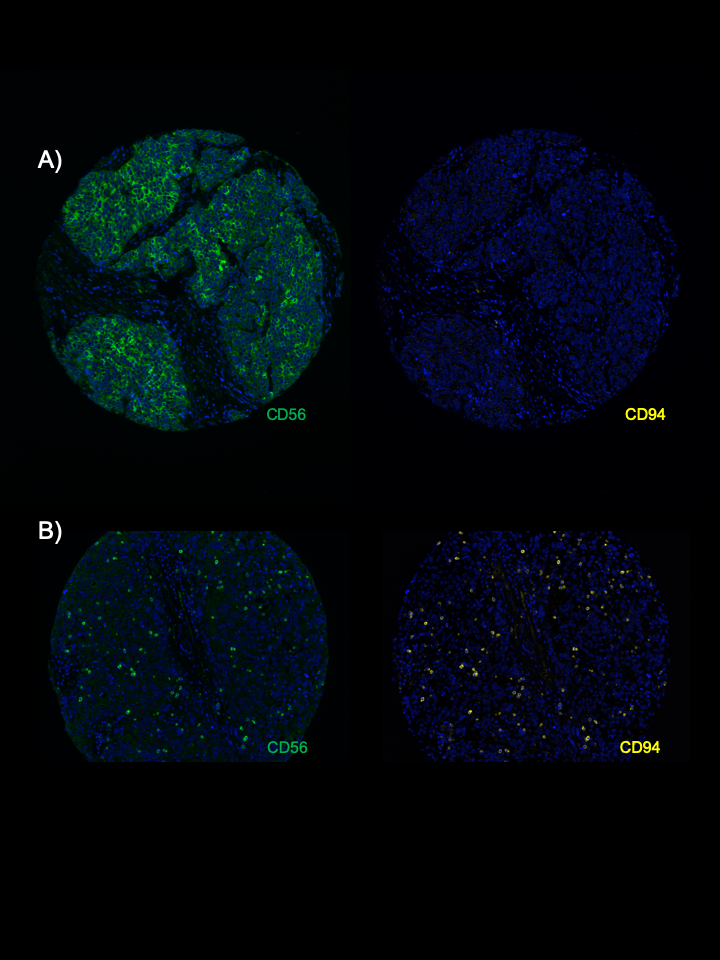
*

**Supplementary Figure 2. CD94 expression accurately marks NK cells in HGSC. A)** CD56 (green) staining on an HGSC where CD56 is overexpressed in the tumor (left) and CD94 (yellow) staining of the same tumor (right). **B)** CD56 staining in an HGSC tumor where CD56 is not expressed on the tumor itself, and superimposable staining of CD94 on the same section. Sections were counterstained with DAPI (blue) and imaged using a PhenoImager Quantitative Pathology workstation at 10x magnification.

| PanCk Pathology Views | InForm Tissue Segmentation | Pathologist Tissue Segmentation | DAPI Pathology Views | InForm Cell Segmentation | InForm Count | Pathologist Count | Cell count accuracy in % |
| --- | --- | --- | --- | --- | --- | --- | --- |
| 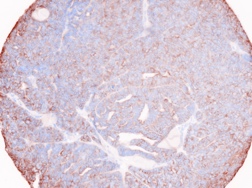 | 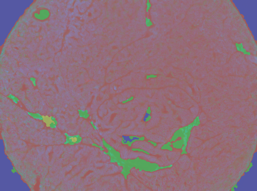 | 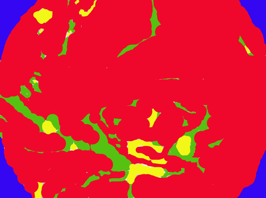 | 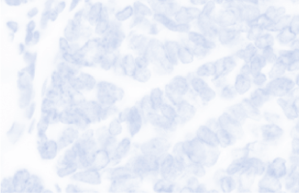 | 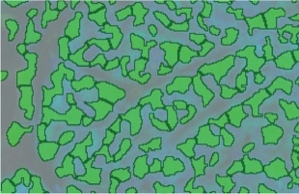 | 119 | 175 | 68% |
| 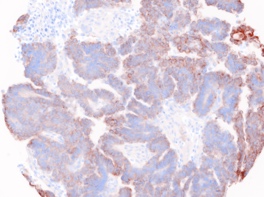 | 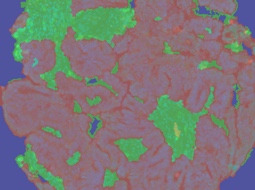 | 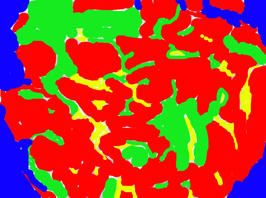 | 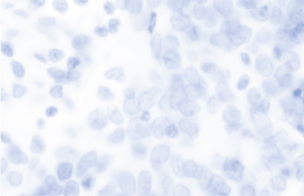 | 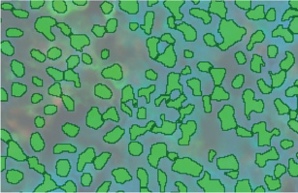 | 130 | 147 | 88% |
| 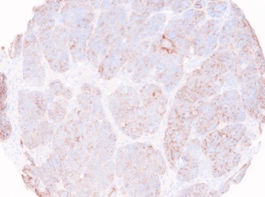 | 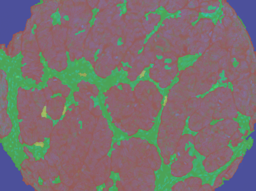 | 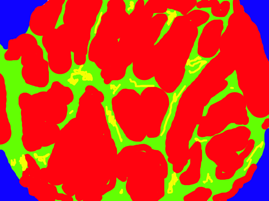 | 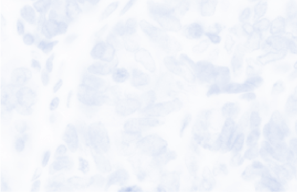 | 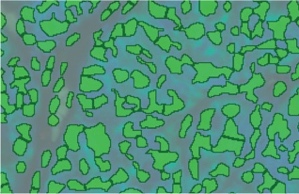 | 143 | 156 | 92% |
| 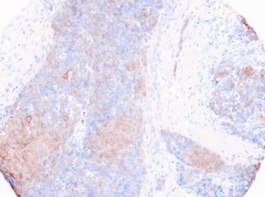 | 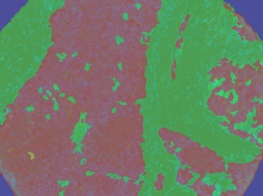 | 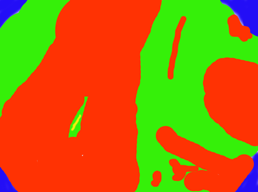 | 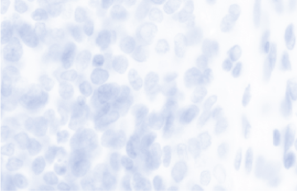 | 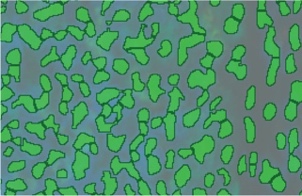 | 135 | 142 | 95% |
| 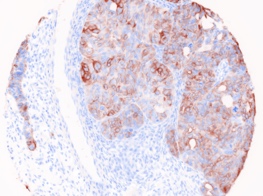 | 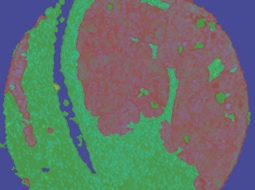 | 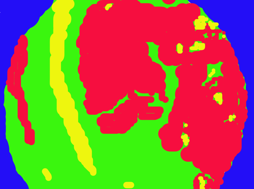 | 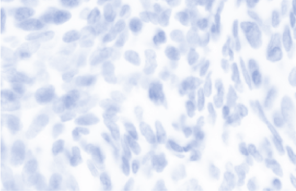 | 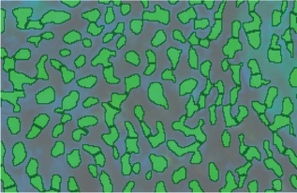 | 143 | 190 | 75% |
| 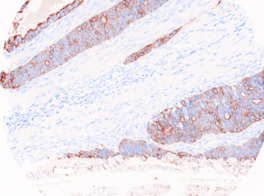 | 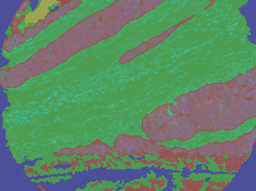 | 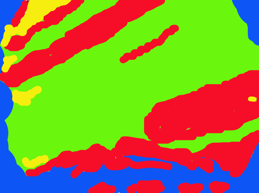 | 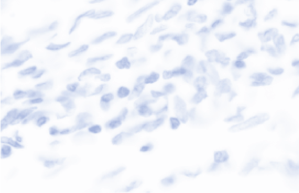 | 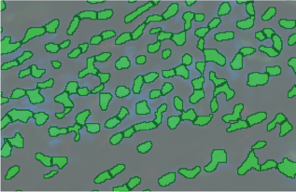 | 105 | 113 | 93% |
| 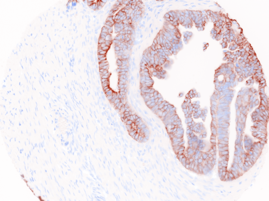 | 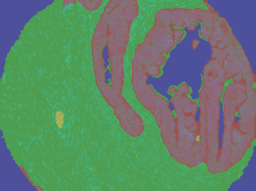 | 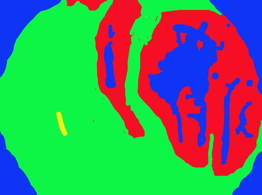 | 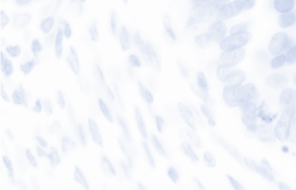 | 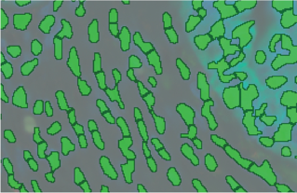 | 116 | 105 | 110% |
| 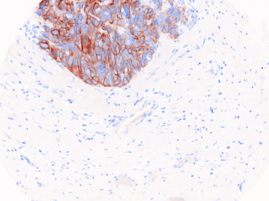 | 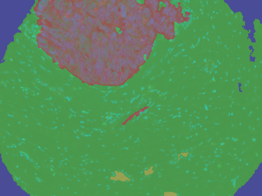 | 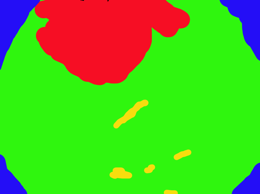 | 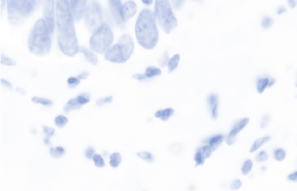 | 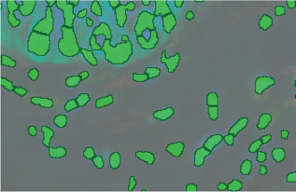 | 71 | 60 | 118% |
| 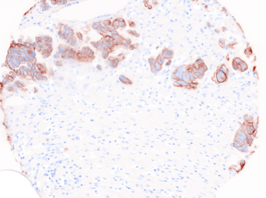 | 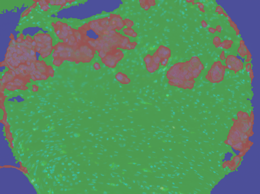 | 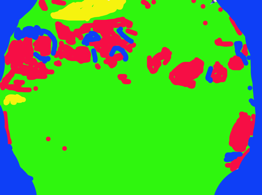 | 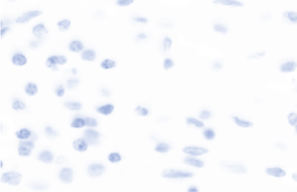 | 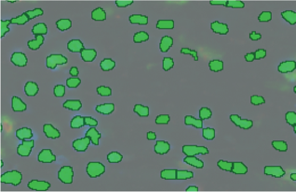 | 70 | 67 | 104% |
| 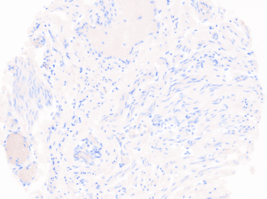 | 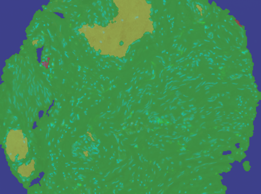 | 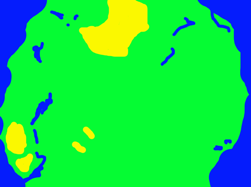 | 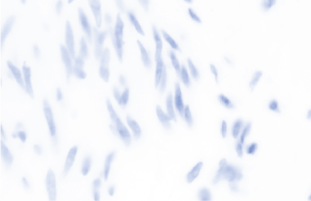 | 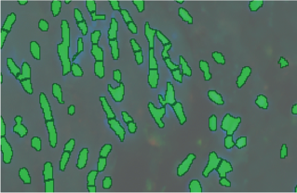 | 83 | 73 | 113% |

**Supplementary Figure 3: Representative digital and manual pathology segmentation, scoring, and pathologist validation.** Tissue segmentation was assigned to each core after training of the machine learning algorithm in InForm digital pathology software based on anti-Pan-cytokeratin staining. Pathologist TA segmented the same cores, blinded to the InForm outcomes. Pathology views (artificial H&E staining), InForm segmentation and the pathologist’s segmentation are shown. Cell segmentation was trained to recognize individual cell nuclei based on DAPI staining, and individual cells were counted by InForm or TA. Computed or counted numbers and the percent accuracy (InForm Count/ Pathologist’s Count) of the computational analysis to match the pathologist’s counts are shown.

**
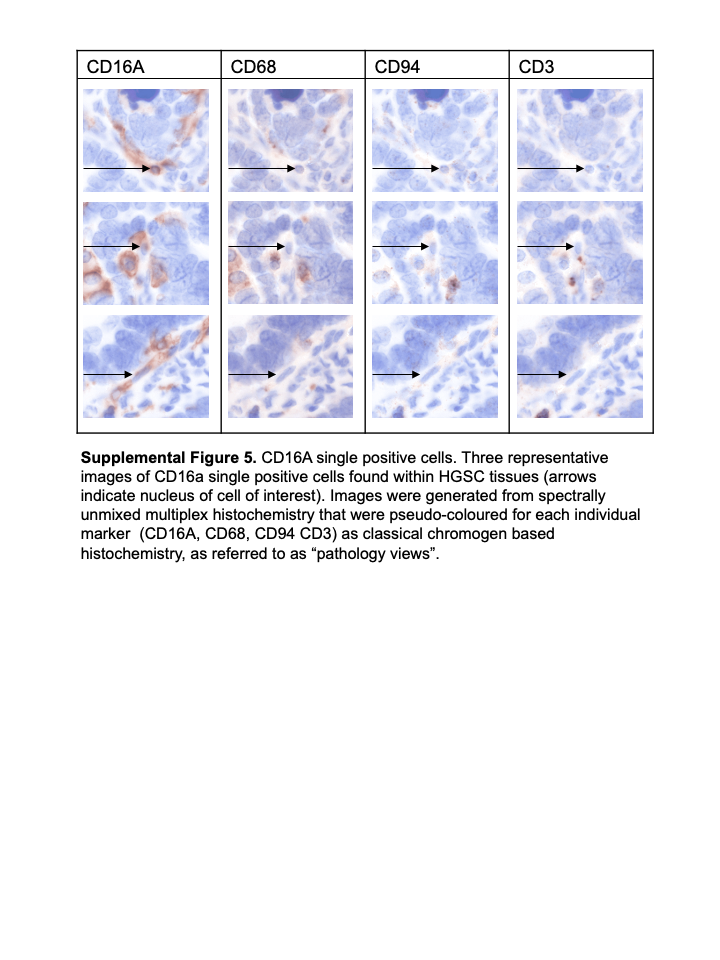
**

**Supplementary Figure 4. CD16A single positive cells.** Three representative images of CD16a single positive cells found within HGSC tissues (arrows indicate nucleus of cell of interest). Images were generated from spectrally unmixed multiplex histochemistry that were pseudo-coloured for each individual marker (CD16A, CD68, CD94 CD3) as classical chromogen based histochemistry, which are referred to as “pathology views” in InForm software.

**
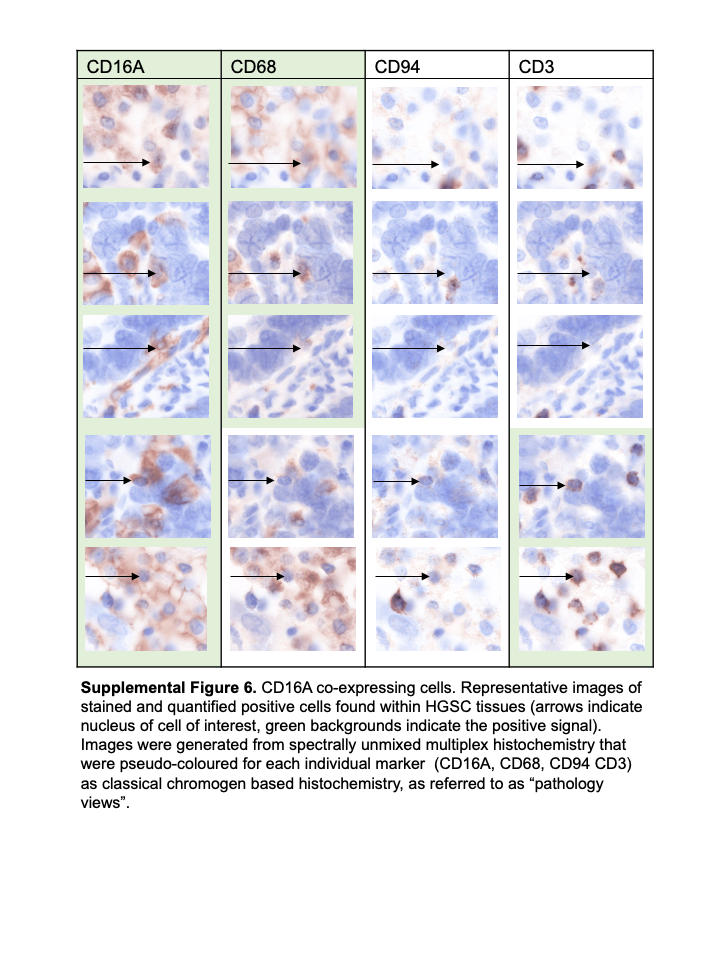
**

**Supplementary Figure 5. CD16A co-expressing cells.** Representative images of stained and quantified positive cells found within HGSC tissues (arrows indicate nucleus of cell of interest, green backgrounds indicate the positive signal). Images were generated from spectrally unmixed multiplex histochemistry that were pseudo-coloured for each individual marker (CD16A, CD68, CD94 CD3) as classical chromogen based histochemistry, as referred to as “pathology views”.

**Supplementary Table 1. Aggregate clinical data for treatment-naïve patients represented on TMA A and TMA B.**

| **Characteristics** | Median (range) | | |
| --- | --- | --- | --- |
| Age | 62 (26-91) | | |
| **Category** | | **N** | **%** |
| Patients | Patients included | 877 | 100 |
| Diagnosis Year | 1992-1995 | 12 | 1 |
|  | 1996-2000 | 75 | 9 |
|  | 2001-2005 | 261 | 30 |
|  | 2006-2010 | 406 | 46 |
|  | 2011-2012 | 95 | 11 |
|  | 2013-2014 | 19 | 2 |
|  | Not reported | 9 | 1 |
| Tumor Grade | Grade 1 | 2 | 0.22 |
|  | Grade 2 | 91 | 10 |
|  | Grade 3 | 675 | 77 |
|  | Not reported | 109 | 12 |
| FIGO Tumor Stage | I | 46 | 5 |
|  | II | 90 | 10 |
|  | III | 621 | 71 |
|  | IV | 88 | 10 |
|  | Not reported | 32 | 4 |
| BRCA Status | BRCA WT | 287 | 33 |
|  | BRCA1 mutated | 41 | 47 |
|  | BRCA2 mutated | 17 | 19 |
|  | BRCA1/2 mutated | 1 | 0.1 |
|  | BRCA mutated (1/2 not specified) | 1 | 0.1 |
|  | Not reported | 530 | 60 |
| Debulking Status | No residual disease | 315 | 36 |
|  | Residual disease | 309 | 35 |
|  | Not reported | 253 | 29 |

| **Supplementary Table 2. OPAL TSA-based multiplex immunofluorescence panels**   \| **Marker** \| **Round** \| **Species** \| **Clone #** \| **Blocking** \| **AR** \| **Polymer** \| **Ab Dilution** \| **OPAL** \| **OPAL Dilution** \| \| --- \| --- \| --- \| --- \| --- \| --- \| --- \| --- \| --- \| --- \| \| CD3 \| **1** \| Mouse \| LN10 \| OPAL \| 9 \| Ms + Rb HRP \| 1/50 \| 650 \| 1/100 \| \| CD16a \| **2** \| Rabbit \| SP175 \| OPAL \| 9 \| Ms + Rb HRP \| 1/50 \| 520 \| 1/100 \| \| CD94 \| **3** \| Rabbit \| ERP21003 \| OPAL \| 9 \| Ms + Rb HRP \| 1/100 \| 570 \| 1/100 \| \| CD68 \| **4** \| Mouse \| PGM1 \| OPAL \| 9 \| Ms + Rb HRP \| 1/100 \| 620 \| 1/100 \| \| panCk \| **5** \| Mouse \| AE1/AE3 \| OPAL \| 9 \| Ms + Rb HRP \| 1/50 \| 480 \| 1/50 \| \| DAPI \| **6** \|  \|  \|  \|  \|  \|  \|  \|  \| \| CD163 \| **1** \| Rabbit \| EPR19518 \| OPAL \| 9 \| Ms + Rb HRP \| 1/100 \| 540 \| 1/100 \| \| CD68 \| **2** \| Mouse \| PGM1 \| OPAL \| 9 \| Ms + Rb HRP \| 1/100 \| 690 \| 1/100 \| \| CD94 \| **3** \| Rabbit \| ERP21003 \| OPAL \| 9 \| Ms + Rb HRP \| 1/50 \| 520 \| 1/100 \| \| panCk \| **4** \| Mouse \| AE1/E3 \| OPAL \| 9 \| Ms + Rb HRP \| 1/50 \| 570 \| 1/100 \| \| CD16a \| **5** \| Rabbit \| SP175 \| OPAL \| 9 \| Ms + Rb HRP \| 1/50 \| 650 \| 1/100 \| \| CD8 \| **6** \| Mouse \| C8/144 \| OPAL \| 9 \| Ms + Rb HRP \| 1 \| 480 \| 1/100 \| \| DAPI \| **7** \|  \|  \|  \|  \|  \|  \|  \|  \| |
| --- | --- | --- | --- | --- | --- | --- | --- | --- | --- | --- | --- | --- | --- | --- | --- | --- | --- | --- | --- | --- | --- | --- | --- | --- | --- | --- | --- | --- | --- | --- | --- | --- | --- | --- | --- | --- | --- | --- | --- | --- | --- | --- | --- | --- | --- | --- | --- | --- | --- | --- | --- | --- | --- | --- | --- | --- | --- | --- | --- | --- | --- | --- | --- | --- | --- | --- | --- | --- | --- | --- | --- | --- | --- | --- | --- | --- | --- | --- | --- | --- | --- | --- | --- | --- | --- | --- | --- | --- | --- | --- | --- | --- | --- | --- | --- | --- | --- | --- | --- | --- | --- | --- | --- | --- | --- | --- | --- | --- | --- | --- | --- | --- | --- | --- | --- | --- | --- | --- | --- | --- | --- | --- | --- | --- | --- | --- | --- | --- | --- | --- | --- | --- | --- | --- | --- | --- | --- | --- | --- | --- |

| **Supplementary Table 3.** Cox proportional hazards analysis for progression-free survival (PFS) and overall survival (OS) for 886 treatment-naïve patients based on clinical characteristics, infiltrating immune cells and area covered by immune cell neighborhoods. | | | | | | |
| --- | --- | --- | --- | --- | --- | --- |
| **Univariate analysis** | **Progression-free survival** | | | **Overall survival** | | |
| **Clinical Variables** | **HR** | **95% CI** | ***p*** | **HR** | **95% CI** | ***p*** |
| Age (older vs. younger patients) | 1.00 | [0.997,1.01] | 0.234 | 1.02 | [1.01, 1.03] | <0.0001 |
| *BRCA1/2* mutation (mutant vs. wildtype) | 0.867 | [0.691, 1.09] | 0.227 | 0.756 | [0.556, 1.03] | 0.0750 |
| Grade (grade 3 vs. grade 2) | 1.09 | [0.847, 1.39] | 0.517 | 1.08 | [0.796,1.46] | 0.624 |
| Stage (high stage vs. low stage) | 1.93 | [1.68, 2.22] | <0.0001 | 1.76 | [1.48, 2.11] | <0.0001 |
|  |  |  |  |  |  |  |
| Debulking status (not debulked vs. successfully debulked) | 2.18 | [1.80, 2.64] | <0.0001 | 2.48 | [1.95, 3.15] | <0.0001 |
| **Immune Infiltration (categorical variable, +/- median)** | | | | | | |
| Stroma Other | 1.01 | [0.856, 1.18] | 0.936 | 0.931 | [0.762, 1.14] | 0.482 |
| Epithelium Other | 0.994 | [0.845, 1.17] | 0.941 | 1.01 | [0.826, 1.24] | 0.926 |
| Stroma PanCK | 1.08 | [0.923, 1.28] | 0.324 | 1.13 | [0.927, 1.39] | 0.221 |
| Epithelium PanCK | 0.992 | [0.844, 1.17] | 0.935 | 0.979 | [0.801, 1.97] | 0.836 |
| Stroma CD16A | 0.922 | [0.784, 1.08] | 0.327 | 0.861 | [0.705, 1.05] | 0.146 |
| Epithelium CD16A | 0.869 | [0.738, 1.02] | 0.0756 | 0.836 | [0.684, 1.02] | 0.0822 |
| Stroma CD68 | 1.08 | [0.920, 1.3] | 0.340 | 0.932 | [0.763, 1.14] | 0.493 |
| Epithelium CD68 | 0.897 | [0.76, 1.0] | 0.190 | 0.856 | [0.700, 1.05] | 0.130 |
| Stroma CD68CD16A | 0.869 | [0.739, 1.02] | 0.0913 | 0.799 | [0.653, 0.978] | 0.0295 |
| Epithelium CD68CD16A | 0.869 | [0.738, 1.02] | 0.0902 | 0.782 | [0.639, 0.958] | 0.0177 |
| Stroma CD3 | 0.987 | [0.840, 1.16] | 0.879 | 1.00 | [0.820, 1.23] | 0.982 |
| Epithelium CD3 | 1.03 | [0.879, 1.22] | 0.689 | 1.04 | [0.849, 1.27] | 0.716 |
| Stroma CD3CD16A | 0.909 | [0.773, 1.16] | 0.250 | 0.916 | [0.749, 1.12] | 0.389 |
| Stroma CD94 | 1.05 | [0.922, 1.27] | 0.326 | 1.02 | [0.834, 1.25] | 0.850 |
| Epithelium CD94 | 0.957 | [0.813, 1.13] | 0.596 | 0.827 | [0.676, 1.01] | 0.0652 |
| Stroma CD94CD16A | 0.973 | [0.827, 1.14] | 0.737 | 0.846 | [0.693, 1.04] | 0.105 |
| **Neighborhood proportion (categorical variable, +/- mean)** | | | | | | |
| Cold epithelium | 0.968 | [0.823, 1.14] | 0.698 | 0.995 | [0.815, 1.22] | 0.964 |
| Cold stroma | 1.05 | [0.897, 1.24] | 0.522 | 1.04 | [0.852, 1.27] | 0.691 |
| Immune rich stroma | 1.04 | [0.883, 1.22] | 0.648 | 1.01 | [0.825, 1.23] | 0.939 |
| Immune moderate | 1.13 | [0.957, 1.32] | 0.154 | 1.09 | [0.895, 1.34] | 0.382 |
| CD16A enriched stroma | 0.850 | [0.723, 1.00] | 0.0499 | 0.850 | [0.695, 1.04] | 0.111 |
| CD16A enriched epithelium | 0.835 | [0.710, 0.982] | 0.0297 | 0.781 | [0.639, 0.955] | 0.0160 |
| **Immune Infiltration (categorical variable, Q1/Q4)** | | | | | | |
| Stroma Other | 1.02 | [0.813, 1.28] | 0.862 | 1.05 | [0.794, 1.39] | 0.735 |
| Epithelium Other | 0.964 | [0.767, 1.21] | 0.757 | 0.780 | [0.586, 1.04] | 0.0883 |
| Stroma PanCK | 1.10 | [0.813, 1.28] | 0.399 | 1.34 | [1.01, 1.78] | 0.0407 |
| Epithelium PanCK | 0.950 | [0.753, 1.20] | 0.662 | 1.08 | [0.814, 1.43] | 0.586 |
| Stroma CD16A | 0.777 | [0.618, 0.977] | 0.0307 | 0.688 | [0.515, 0.920] | 0.0115 |
| Epithelium CD16A | 0.802 | [0.636, 1.01] | 0.0645 | 0.753 | [0.563, 1.01] | 0.0556 |
| Stroma CD68 | 1.11 | [0.881, 1.39] | 0.385 | 0.981 | [0.739, 1.30] | 0.895 |
| Epithelium CD68 | 0.990 | [0.786, 1.25] | 0.934 | 0.799 | [0.601, 1.06] | 0.125 |
| Stroma CD68CD16A | 0.828 | [0.657, 1.05] | 0.113 | 0.715 | [0.539, 0.948] | 0.0199 |
| Epithelium CD68CD16A | 0.984 | [0.781, 1.24] | 0.893 | 0.866 | [0.649, 1.16] | 0.328 |
| Stroma CD3 | 1.02 | [0.810, 1.29] | 0.854 | 1.08 | [0.812, 1.45] | 0.588 |
| Epithelium CD3 | 1.04 | [0.839, 1.29] | 0.714 | 1.13 | [0.867, 1.48] | 0.362 |
| Stroma CD3CD16A | 0.918 | [0.748, 1.13] | 0.416 | 0.879 | [0.678, 1.14] | 0.327 |
| Epithelium CD3CD16A [1 vs 3]* | 0.925 | [0.756, 1.13] | 0.451 | 0.870 | [0.672, 1.13] | 0.290 |
| Stroma CD94 | 1.13 | [0.925, 1.39] | 0.227 | 1.04 | [0.807, 1.34] | 0.756 |
| Epithelium CD94 | 0.947 | [0.773, 1.16] | 0.598 | 0.823 | [0.637, 1.06] | 0.137 |
| Stroma CD94CD16A | 0.960 | [0.785, 1.18] | 0.692 | 0.798 | [0.615, 1.03] | 0.0881 |
| Epithelium CD94CD16A [1 vs 2]* | 1.06 | [0.854, 1.32] | 0.595 | 1.10 | [0.837, 1.44] | 0.497 |
| **Neighborhood proportion (categorical variable, +/- mean) (n=24)** | | | | | | |
| N0 | 2.05 | [0.814, 5.16] | 0.128 | 1.60 | [0.551, 4.64] | 0.388 |
| N1 | 1.39 | [0.556, 3.47] | 0.482 | 1.26 | [0.436, 3.64] | 0.670 |
| N2 | 0.477 | [1.88, 1.21] | 0.119 | 1.04 | [0.364, 2.97] | 0.942 |
| N3 | 0.829 | [0.334, 2.06] | 0.687 | 0.826 | [0.289, 2.36] | 0.720 |
| N4 | 0.532 | [0.213, 1.33] | 0.176 | 0.389 | [0.130, 1.17] | 0.0921 |
| N5 | 0.530 | [0.210, 1.34] | 0.179 | 0.866 | [0.303, 2.48] | 0.788 |
| N6 | 0.588 | [0.238, 1.45] | 0.250 | 0.242 | [0.0748, 0.785] | 0.0181 |
| N7 | 1.19 | [0.480, 2.93] | 0.712 | 0.682 | [0.236, 1.97] | 0.479 |

*due to low cell densities across tumor cores, four quartiles were not identified and the hazard model was performed with available groups indicated.
